# The Consequences of A History of Violence on Women’s Pregnancy and Childbirth in the Nordic Countries: A Scoping Review

**DOI:** 10.1177/15248380241253044

**Published:** 2024-05-28

**Authors:** Hafrún Rafnar Finnbogadóttir, Lena Henriksen, Hanne Kristine Hegaard, Sigridur Halldórsdóttir, Eija Paavilainen, Mirjam Lukasse, Lotte Broberg

**Affiliations:** 1 Linnaeus University, Växjö/ Kalmar, Sweden; 2 Oslo Metropolitan University, Norway; 3 Copenhagen University Hospital-Rigshospitalet, Denmark; 4 The University of Copenhagen, Denmark; 5 University of Akureyri, Iceland; 6 Tampere University/Etelä-Pohjanmaa Hospital District, Finland; 7 University of Southeastern Norway, Kongsberg, Norway; 8 Bispebjerg and Frederiksberg Hospital, Denmark; 9 Slagelse Hospital, Denmark

**Keywords:** childbirth, domestic violence, history of violence, pregnancy, perinatal period, Nordic countries

## Abstract

Violence against women (VAW) is a global challenge also in the childbearing period. Despite high gender equality, there is a high prevalence of VAW in the Nordic countries. This scoping review aims to explore predictors for and consequences of a history of violence on women’s pregnancy and childbirth in the Nordic countries, including women’s experience of the impact of violence and the interventions used to detect, address consequences, and prevent further violence. The framework by Arksey and O’Malley was followed, and English, Finnish, Icelandic, Norwegian, Danish, and Swedish literature was included. The population was women aged ≥18 residing in the Nordic countries during the perinatal period. Eight databases were searched: MEDLINE, CINAHL, PubMed, PsycINFO, Web of Science, ASSIA, Social Services-, and Sociological abstracts. There was no limitation of the search time frame. The initial screening resulted in 1,104 records, and after removing duplicates, 452 remained. Finally, 61 papers met the inclusion criteria. The results covering the past 32 years indicated that childbearing women with a history of violence are at greater risk of common complaints and hospitalization during pregnancy, fear of childbirth, Cesarean section, breastfeeding difficulties, and physical and mental health problems. While extensive research was found on the associations between a history of and current violence and outcomes related to pregnancy, there was a lack of intervention studies and studies from Finland. Efforts must be made to scientifically test the methods used to reduce and treat the adverse effects of a history of violence and prevent further violence.

Violence against women (VAW) is a universal public health concern (World Health Organization (WHO), 2021). According to the WHO, psychological, physical, or sexual intimate partner violence (IPV) is the most widespread form of VAW globally (Sardinha et al., 2022), mainly occurring in the woman’s own home (Garcia-Moreno et al., 2006). The WHO multi-country study reported that in 90% of abused childbearing women, the perpetrator was the unborn child’s biological father (WHO, 2005). VAW endangers maternal and fetal health (Donovan et al., 2016; Hill et al., 2016), leading to poorer physical and mental health (Alhusen et al., 2015; Hill et al., 2016; Howard et al., 2013), also seen in a life perspective (Rivara et al., 2019).

In the Nordic countries, the prevalence of a history of violence (lifetime experience of emotional, physical, or sexual abuse) is between 33.6% and 39.5% among pregnant women (Finnbogadóttir et al., 2014a; Lukasse et al., 2014). The reporting of any violence occurring during pregnancy is much lower, with 8.5% of pregnant women who report violence in Denmark (Andreasen et al., 2023), 5% in Norway (Sørbø et al., 2013), 3.3% in Iceland prenatally and during pregnancy (Lukasse et al., 2014), and 2.5% in Sweden during pregnancy (Finnbogadóttir & Dykes, 2016). No data were found for the prevalence of exposure to violence during pregnancy in Finland.

The “Nordic paradox” denotes comparatively high levels of VAW in countries estimated to be the most equal globally, such as Nordic countries (Gracia & Merlo, 2016). The Gender Equality Index comprises six core domains: work, money, knowledge, time, power, and health. Finland, Iceland, Norway, and Sweden are ranked in the top ten places regarding gender equality, with Iceland in first place and Denmark in place fourteen (World Economic Forum, 2022). Therefore, differences in the sociodemographic characteristics or gender inequality at the country level do not seem to explain the high prevalence of VAW. The Nordic countries share a common history and culture, a similar healthcare system, and close political cooperation, making collaborative efforts particularly valuable for childbearing women and their children. Gaining a better understanding of the predictors for, consequences, and potential preventive strategies for gender-based violence in the Nordic population is important. It can contribute to a deeper understanding of the complex interplay between sociocultural factors, health care systems, and VAW in diverse contexts worldwide. Therefore, this scoping review aims to explore predictors for and consequences of a history of violence on women’s pregnancy and childbirth in the Nordic countries, including women’s experience of the impact of violence and the interventions used to detect, address consequences, and prevent further violence.

## Research Questions

1. How do women experience and describe the impact of violence on their pregnancy and childbirth?
2. What are the predictors of violence exposure during the perinatal period?
3. What is the association between a history of violence and physical outcomes?
4. What is the association between a history of violence and psychological outcomes?
5. What is the association between a history of violence and obstetric outcomes?
6. What interventions are used to detect violence, address the consequences, and prevent further violence?

## Method

A scoping review was chosen to map existing literature and identify research gaps. The scoping review was conducted according to the methodological framework outlined by Arksey and O’Malley that consists of five consecutively linked stages: (a) identifying the research question, (b) identifying relevant studies, (c) selecting studies, (d) charting the data, and (e) collating, summarizing, and reporting the results (O’Mallay, 2005). The guidelines of Peters and the Joanna Briggs Institute (JBI) were also followed (Peters et al., 2020). The review is reported according to the Preferred Reporting Items for Systematic Reviews and Meta-Analyses Extension for Scoping Reviews (PRISMA-ScR) (“PRISMA Extension for Scoping Reviews (PRISMA-ScR): Checklist and Explanation”, 2018).

### Inclusion and Exclusion Criteria

The inclusion of studies was based on the Population-Concept-Context framework, as recommended by JBI (Peters et al., 2020), to align the study selection with the research questions. The population of interest consisted of women aged 18 or older who were either pregnant or had given birth within the previous year and were living in the Nordic countries: Finland, Iceland, Norway, Denmark (Faroe Islands and Greenland included), or Sweden.

### Definitions

The *perinatal period* is defined as a period when a woman becomes pregnant and up to 1 year after giving birth.

*History of violence* is defined as a lifetime experience of emotional, physical, or sexual abuse without consideration of the level of abuse or the perpetrator’s identity (Finnbogadóttir et al., 2014a).

*Domestic violence* (DV) is defined as physical, sexual, psychological, mental, or emotional violence or threats of physical or sexual violence that is inflicted on a woman by a family member, such as a marital or cohabiting partner, parents, siblings, or a person very well known in the family or a significant other, often taking place in the home (Krug, 2002).

*IPV* refers to the same action described above for DV undertaken by an intimate partner (male/female), such as a marital or cohabiting partner (Krug, 2002).

### Types of Literature

Articles with quantitative, qualitative, or mixed methods designs were included if the research had been conducted in the Nordic countries and met the inclusion criteria.

Relevant gray literature included reports, policy literature, guidelines, working papers, and government documents. Only literature written in languages the research team could read and understand was included. These languages are English, Finnish, Icelandic, Norwegian, Danish, and Swedish.

### Search Strategy

Eight databases (MEDLINE, CINAHL, PubMed, PsycINFO, WoS, ASSIA, social services abstracts, and sociological abstracts) were searched on March 13, 2022, by a librarian (IH) of the Faculty of Health and Life Sciences, Linnaeus University, Sweden. A broad search was performed following stages one and two of Arksey O’Malley’s methodology (O’Mallay, 2005). The literature search was developed in a collaboration between the IH at Linnaeus University and the first author (HRF). It was decided that the literature search should consist of the following elements: (a) The geographical area that encircles the Nordic region, (b) the perinatal period, and (c) a history of DV and IPV. There was no time limitation for the literature search, including literature written in English, Finnish, Icelandic, Norwegian, Danish, and Swedish. The complete search strategy can be found in supplementary material (Supplemental material Appendix A). In addition, the researchers from each of the five countries, namely Finland, Iceland, Norway, Denmark (Faroe Islands and Greenland included), and Sweden, searched for gray literature. Reference lists in the already included articles were checked, and key journals, existing networks, relevant organizations, and conference contributions were manually searched. Additionally, a Google Scholar search was conducted.

### Data Extraction, Charting Data, and Analysis

**Figure 1.**
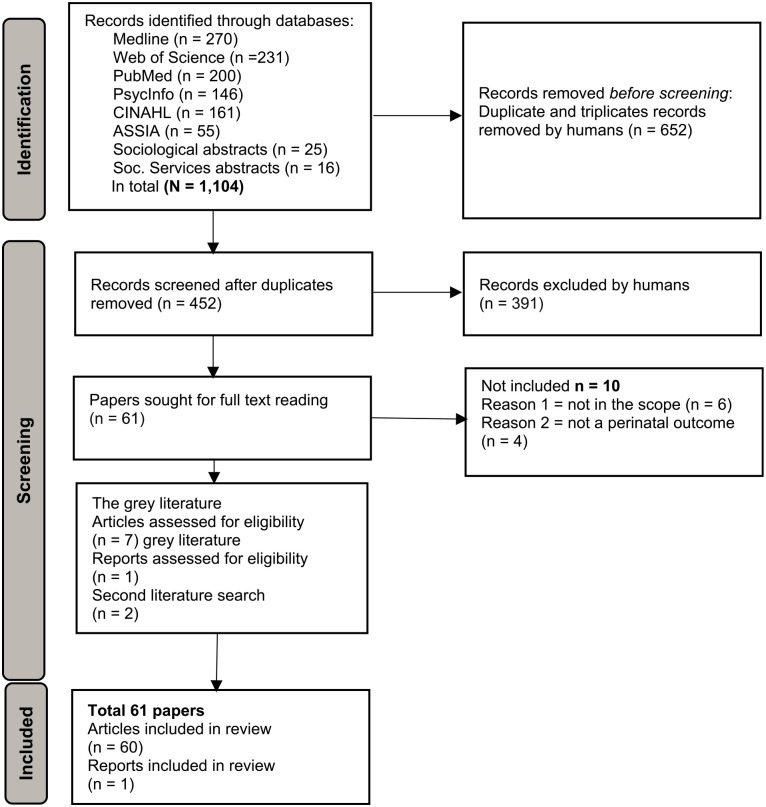
Preferred Reporting Items for Systematic Reviews and Meta-Analyses flowchart. *Note.* Page et al. (2021).

In the initial screening, all search results (*n* = 1,104) were imported into the reference management software (EndNote Version 20, Clarivate, London England), and duplicates were removed by the first author (HRF). All the titles and abstracts (*n* = 452) were imported into Rayyan QCRI and assessed independently by two researchers (HRF and LH). Papers about which there was doubt were discussed until a mutual agreement was reached. This process yielded 61 papers. Full-text versions of the papers were obtained and assessed by four teams, each comprising two independent reviewers. All authors were involved in the extraction process. At least two authors mastered all the Nordic languages, but Finnish, where only one author mastered it. If a reviewer had authored any of the articles, a different team handled the review of that article. An extraction form was developed to assess each paper (Supplemental Material Appendix B). In cases (*n* = 15) where the first team was unsure about including a paper, another team conducted a second assessment. Finally, 49 articles, one editorial letter, and one short communication met the study’s aim and inclusion criteria. The gray literature from each country generated eight more papers: five articles from Norway, one from Iceland, one from Sweden, and a report from Denmark. The first author (HRF) and the last author (LB) read the full text of all included papers and charted the data (Supplemental material Tables S1–S3). One article was excluded during the process of full-text reading because it did not comply with the inclusion criterion of the perinatal period (Supplemental material Table S4). The qualitative data included one peer-reviewed article in Icelandic, and the two Icelandic authors (HRF and SH) extracted, charted, and analyzed the qualitative data (Supplemental material Table S3) using directed content analysis (Hsieh & Shannon, 2005). The search was updated in all databases on April 3, 2023, resulting in two included articles. Some days after the second search, one article was published and replaced the report in Swedish (gray literature). Ultimately, data from 61 papers with approximately 171,500 participants were included in the scoping review (Figure 1).

## Findings

A total of 61 papers published between 1991 and 2023 were included. Of these, 35 articles presented data from Norway, including data from four articles from a multi-country study including Denmark, Iceland, and Sweden. Seventeen articles from Sweden were included, including one from the above-mentioned multi-country study. The scoping review also included three articles and one report from Denmark, four from Iceland, and one from Finland. At least 60% of the included papers have reported immigrant women as a part of the investigated population (Supplemental material Tables S1–S3).

The study designs were cohort studies (*n* = 17), case-control studies (*n* = 9), a randomized controlled study (RCT) (*n* = 1), a nested cohort intervention study (*n* = 1), a letter to the editor (*n* = 1) (Supplemental material Table S1), cross-sectional studies (*n* = 27) (Supplemental material Table S2), qualitative studies (*n* = 4) (Supplemental material Table S4), and one report from Denmark (SocialRespons, 2022). For an overview of all included articles, see Table 1.

**Table 1.** Characteristics of Included Studies.

First Author (Year)	Origin	Design and Sample	Aim
Andreasen (2023)	Denmark	A cohort intervention study *N* = 54	To assess the feasibility of implementing digital intimate partner violence (IPV) screening among pregnant women and a digital supportive intervention targeting pregnant women exposed to IPV
Drevin (2020)	Norway	A cross-sectional study *N* = 76.197	To study if childhood emotional, physical, and sexual abuse are determinants for having an unplanned pregnancy
Eberhard-Gran (2008)	Norway	A cross-sectional study *N* = 414	To study the occurrence of extreme fear during labor and its association with previous sexual abuse in adult life
Edin (2010)	Sweden	Qualitative Descriptive study *N* = 9	To explore how women from different ethnic backgrounds experienced intimate partner violence (IPV) and their recommendations for antenatal care
Eide (2010)	Norway	A cross-sectional study *N* = 60,775	To explore the association between sexual and/or physical abuse in childhood and worries about the baby’s health in pregnancy
Eikemo (2023)	Sweden	A cross-sectional study *N* = 3,399	To describe the prevalence of physical, psychological, and sexual IPV among pregnant women and investigate potential associations between exposure and sociodemographic characteristics and health
Engnes (2012)	Norway	Phenomenological study *N* = 5	To enter deeply into the experience of living with violence during pregnancy
Finnbogadóttir (2011)	Sweden	A population-based multicenter cohort study *N* = 2,652	To investigate whether self-reported history of violence or experienced violence during pregnancy is associated with increased risk of labor dystocia in nulliparous women at term
Finnbogadóttir (2014a)	Sweden	A cross-sectional study *N* = 1,939	To explore prevalence of domestic violence (DV) among pregnant women and associations with background factors
Finnbogadóttir (2014b)	Sweden	Grounded theory approach *N* = 10	To develop a grounded theoretical model of women’s experience of IPV during pregnancy and how they handle their situation
Finnbogadóttir (2016a)	Sweden	A longitudinal cohort study *N* = 1,509	To explore the prevalence and incidence of DV among pregnant women and their experience of a history of violence and explore the association between DV during pregnancy and possible risk factors
Finnbogadóttir (2016b)	Norway	A longitudinal cohort study *N* = 731	To explore the prevalence and incidence of DV during pregnancy and postpartum and the history of violence among new mothers
Finnbogadóttir (2017a)	Sweden	A cross-sectional study *N* = 1,939	To explore the degree of self-reported suffering following violent incidents and the prevalence of police reporting and other help-seeking behavior among women in early pregnancy with a history of violence
Finnbogadóttir (2017b)	Sweden	A cross-sectional study *N* = 731	To determine the differences in breastfeeding among women who did and did not experience DV during pregnancy and postpartum in a Swedish context
Finnbogadóttir (2020)	Sweden	A longitudinal cohort study *N* = 1,939	To explore childbirth outcomes in a Swedish population of women reporting a history of violence, including DV during pregnancy
Flaathen (2021)	Norway	A prospective cross-sectional study *N* = 1,788	To investigate the association between unintended pregnancy and emotional, physical, and sexual IPV in a multi-cultural population attending routine antenatal care
Flaathen (2022)	Norway	A multicenter randomized controlled study *N* = 1,818	To examine if the safe pregnancy intervention would prevent IPV and positively affect women’s health and quality of life
Gísladóttir (2014)	Iceland	Register-based prospective cohort study *N* = 1,068	To determine whether women exposed to sexual violence in adolescence or adulthood are at increased risk of adverse maternal characteristics during subsequent pregnancies
Gísladóttir (2016)	Iceland	A case-control study *N* = 10,077	To investigate whether women exposed to sexual violence as teenagers or adults present different obstetric outcomes than women with no record of such violence
Gissler (1999)	Finland	Letter to editor, a register study *N* = 9,192	Pregnancy-related violent deaths. Study aim: not described
Grimstad (1997)	Norway	A case-control study *N* = 178	To investigate the association between physical abuse and low birthweight
Grimstad (1998)	Norway	A case-control study *N* = 178	To study the relationship between a history of physical abuse and consumption of cigarettes and alcohol during pregnancy
Grimstad (1999a)	Norway	A case-control study *N* = 177	To assess the relationships among anxiety, history of abuse, and low birth weight (LBW)
Grimstad (1999b)	Norway	A case-control study *N* = 174	To re-analyze the data to examine the relation between abuse and birth weight, using a broad and comprehensive definition of abuse
Grimstad (1999c)	Norway	A case-control study *N* = 178	To study whether the association between a history of child sexual abuse, the risk LBW, smoking habits, obstetric complications, and complaints during pregnancy
Hedin (1999)	Sweden	A prevalence study *N* = 207	To measure the prevalence, effects, and character of psychological abuse in women visiting antenatal clinics and how different forms of abuse correlate
Heimstad (2006)	Norway	A Cross-sectional study *N* = 1,452	To assess the prevalence of fear of childbirth (FOC) and find possible associations to selected sociodemographic factors and important life events
Henriksen (2013)	Norway	A population-based cohort study *N* = 78,660	To investigate if a history of sexual violence is associated with hospitalization during pregnancy
Henriksen (2014a)	Norway	A populations-based cohort study *N* = 74,059	To explore the association between sexual violence and mode of delivery
Henriksen (2014b)	Norway	A populations-based cohort study *N* = 76,870	To explore the association between sexual violence and neonatal outcomes
Henriksen (2016)	Norway	A populations-based cohort study *N* = 78,660	To explore the association between lifetime sexual violence and expectations about childbirth
Henriksen (2017)	Norway	A mixed-method study *N* = 1,352	To explore factors associated with a negative childbirth experience, including descriptions from women
Knoph Berg (2011)	Norway	A cross-sectional study *N* = 45,644	To identify factors associated with the incidence and course of broadly defined binge eating disorder in pregnancy
Kristinsdóttir (2010)	Iceland	Phenomenological approach *N* = 12	To increase the knowledge and deepen the understanding of DV during pregnancy and at other times
Lukasse (2009)	Norway	A population-based cross-ectional cohort study *N* = 55,776	To examine the prevalence of emotional, physical, and sexual childhood abuse and its association with common complaints in pregnancy
Lukasse (2010a)	Norway	A population-based cross-sectional cohort study *N* = 2,365	To explore the association between self-reported history of sexual, physical, and emotional abuse in childhood and FOC
Lukasse (2010b)	Norway	A population-based cross-sectional cohort study *N* = 26,923	To assess if there is an association between self-reported exposure to sexual, physical, and emotional childhood abuse and birth by cesarean
Lukasse (2011)	Norway	A longitudinal cohort study *N* = 4,876	To examine the association between childhood abuse and FOC and the wish for Cesarean section (CS) during the second pregnancy
Lukasse (2012)	Norway	A population-based national cohort study *N* = 78,660	To examine the association between sexual violence and the physical symptoms during pregnancy
Lukasse (2014a)	Norway	A cross-sectional study *N* = 6,870	To assess the prevalence of severe FOC in six Northern European countries and the association between severe FOC and selected background variables. Finally, to explore if the content of fear was different for the participating countries
Lukasse (2014b)	Norway	A prospective cohort study *N* = 7,200	To investigate the prevalence of a history of abuse among women attending routine antenatal care in six northern European countries and explore current suffering from reported abuse
Lukasse (2015)	Norway	A multi-country cross-sectional study *N* = 7,102	To study the prevalence of unintended pregnancy in six European countries among pregnant women attending routine antenatal care and to investigate the association with a history of physical, sexual, and emotional abuse
Melby (2022)	Norway	A cross-sectional study *N* = 1,812	To investigate the prevalence of antenatal depression and the association between symptoms of antenatal depression and physical, emotional, and sexual abuse in a culturally diverse population attending antenatal care
Nerum (2010)	Norway	A case-control study *N* = 200	To compare the duration of labor and the birth outcome in a group of primiparous women who had been raped after the age of 16 with a control group from the same birth cohort
Nerum (2013)	Norway	A case-control study *N* = 373	To compare the duration and outcome of the first labor in women who have been subjected to childhood sexual abuse and women who have been raped in adulthood
Persson (2020)	Sweden	A cross-sectional study *N* = 945	To estimate the prevalence of potentially traumatic events, FOC, and support for it, as well as posttraumatic stress disorder (PTSD) among pregnant women attending maternal care in Stockholm
Ryding (2015)	Sweden	Longitudinal cohort study *N* = 6,422	To assess the association between FOC and CS in North European women
Schei (1991)	Norway	A cross-sectional cohort study *N* = 306 pregnancies	To assess if there is a relationship between adverse pregnancy outcomes and life in a conjugal relationship characterized by physical abuse
Schei (2014)	Norway	A Cross-sectional cohort study *N* = 6,724	First, to assess whether a history of abuse reported during pregnancy was associated with an operative delivery. Second, to assess if the association varied according to the type of abuse and if the reported abuse had been experienced as a child or an adult
Schroll (2011)	Denmark	A cohort study *N* = 2,638	To estimate the prevalence of self-reported lifetime violence and to assess whether women exposed to any physical violence or sexual violence had a higher risk of having FOC before, during, or after delivery compared with women without such history
Svavarsdóttir (2008)	Iceland	A cross-sectional study *N* = 208	To examine the effects of physical, sexual, and emotional abuse on physical and psychological health among Icelandic women visiting an emergency department or high-risk prenatal care clinic
Stensson (2001)	Sweden	A cohort study *N* = 1,038	To determine the prevalence of current and previous violent acts against pregnant women with an emphasis on physical violence by a close acquaintance or relative, to assess differences between abused and non-abused women regarding socioeconomic characteristics, reproductive and general health problems, pregnancy complications and outcomes
Størksen (2015)	Norway	A population-based cohort study *N* = 1,789	To investigate the demographic and psychological characteristics associated with FOC and the relative importance of such fear as a predictor of elective CS
Sørbo (2014)	Norway	A population-based Cohort study *N* = 53,065	To assess the association between adult abuse and postpartum depression. Secondly, we wanted to explore whether the associations differed if the perpetrator was known or a stranger to the woman
Sørbo (2015)	Norway	A population-based Cohort study *N* = 53,934	To examine whether exposure to past and recent emotional, sexual, or physical abuse was associated with early breastfeeding cessation and to assess whether a potential association differed for known and unknown perpetrators
Tinglöf (2015)	Sweden	A cross-sectional study *N* = 1,514	To examine lifetime exposure to violence, physical and sexual, among women seeking termination of pregnancy and its association with sociodemographic factors, PTSD, symptoms of anxiety and depression
Vatnar (2011)	Norway	A cross-sectional study *N* = 157	To assess if there is an association between interactional aspects of IPV during pregnancy and mothers’ perceptions of negative consequences to the fetus or the newborn baby
Vederhus (2022)	Norway	A cross-sectional study *N* = 295 women	To investigate the prevalence of self-reported emotional, physical, and sexual abuse and its association with FOC in pregnant women with epilepsy
Wangel (2016)	Sweden	A cross-sectional study *N* = 1,003	To describe the prevalence of emotional, physical, and sexual abuse and analyze associations with symptoms of depression and posttraumatic stress in pregnancy, by ethnic background
Wikman (2020)	Sweden	A population-based longitudinal cohort study *N* = 2,466	To describe the characteristics related to background and lifestyle, pregnancy, delivery, and postpartum of different perinatal depression trajectories defined according to the onset of depressive symptoms

### Women’s Experience of the Impact of Violence on Their Pregnancy and Childbirth

The women described the violence as highly stressful. They revealed how their focus had been on survival and protecting their unborn babies by adapting to the perpetrators’ will and avoiding confrontation (Edin et al., 2010; Engnes et al., 2012; Finnbogadottir et al., 2014b; Kristinsdóttir & Halldórsdóttir, 2010). One reason for staying was feeling stuck in the relationship and their pregnant body and unable to find a way out (Finnbogadottir et al., 2014b; Kristinsdóttir & Halldórsdóttir, 2010). Consequently, social isolation, loneliness, and a sense of shame in their choice of partner played a role (Edin et al., 2010; Engnes et al., 2012; Finnbogadottir et al., 2014b; Kristinsdóttir & Halldórsdóttir, 2010). Daily life was also characterized by fear, unhappiness, sadness, fatigue, and powerlessness, and trust issues and intense feelings of rejection were common (Edin et al., 2010; Engnes et al., 2012; Finnbogadottir et al., 2014a; Kristinsdóttir & Halldórsdóttir, 2010). The women described feeling controlled, oppressed, and worthless in their relationships and experiencing a lack of consideration sexually, and even described occurrences of violent sex (Edin et al., 2010; Engnes et al., 2012; Finnbogadottir et al., 2014b; Kristinsdóttir & Halldórsdóttir, 2010). The experience was that the IPV increased during the pregnancy (Finnbogadottir et al., 2014b; Kristinsdóttir & Halldórsdóttir, 2010) and that the perpetrator’s behavior jeopardized the safety of the women and the unborn child (Edin et al., 2010; Engnes et al., 2012; Finnbogadottir et al., 2014b; Kristinsdóttir & Halldórsdóttir, 2010). The women related that the destructive relationship had consequences, such as physical symptoms, eating disorders, deep depression, and anxiety (Edin et al., 2010; Engnes et al., 2012; Finnbogadottir et al., 2014b; Kristinsdóttir & Halldórsdóttir, 2010).

Among a smaller share of 100 women living in shelters in Denmark, some described pregnancy and childbirth as protective factors. By contrast, others experienced it as triggering or escalating violence. They expressed that the perpetrator often used the woman’s fear of losing the child as a means of control and manipulation (SocialRespons, 2022).

### Associated Factors and Predictors of Being Exposed to Violence During the Perinatal Period

Studies from Sweden showed that a history of violence was the most decisive single risk factor associated with DV during pregnancy (Finnbogadóttir & Dykes, 2016; Finnbogadóttir et al., 2016).

The following sociodemographic risk factors for being exposed to violence during the perinatal period have been identified in studies from Iceland, Norway, Sweden, and Denmark: younger age (Gisladottir et al., 2014; Grimstad et al., 1999b; Henriksen et al., 2014a; Lukasse et al., 2010b; Schroll et al., 2011), low educational level (Eikemo et al., 2023; Finnbogadóttir et al., 2011; Lukasse et al., 2010b; Schei et al., 1991; Schroll et al., 2011; Tinglöf et al., 2015), being unemployed (Eikemo et al., 2023; Finnbogadóttir, Baird et al., 2020; Finnbogadóttir et al., 2014a; Gisladottir et al., 2014; Hedin & Janson, 1999; Henriksen et al., 2014a; Lukasse et al., 2010b; Nerum et al., 2010), facing financial distress (Finnbogadóttir, Baird et al., 2020), being single or not cohabiting (Eikemo et al., 2023; Finnbogadóttir, Baird et al., 2020; Finnbogadóttir & Dykes, 2016; Finnbogadóttir et al., 2014a, 2016; Gisladottir et al., 2014; Grimstad et al., 1999b; Henriksen et al., 2014a; Lukasse et al., 2010b; Nerum et al., 2010; Schroll et al., 2011; Tinglöf et al., 2015; Wikman et al., 2020), living with a partner and child/children (Eikemo et al., 2023), and being born outside the Nordic countries (Eikemo et al., 2023; Finnbogadóttir et al., 2014a).

The following lifestyle factors during pregnancy were identified in several studies as predicting factors for being exposed to violence during the perinatal period: smoking (Finnbogadóttir, Baird et al., 2020; Gisladottir et al., 2014; Grimstad et al., 1998; Grimstad et al., 1997; Henriksen et al., 2014a; Nerum et al., 2010; Schroll et al., 2011; Tinglöf et al., 2015), alcohol consumption (Grimstad et al., 1998; Henriksen et al., 2014a; Schroll et al., 2011; Tinglöf et al., 2015), medicine use (Schroll et al., 2011), illicit drug use (Gisladottir et al., 2014), and having a pre-pregnancy BMI ≥30 kg/m^2^ was all associated with being exposed to violence during pregnancy (Gisladottir et al., 2014; Henriksen et al., 2014a; Lukasse et al., 2010b; Nerum et al., 2010) and may be seen as sequelae of abuse.

Having an unplanned pregnancy (Schroll et al., 2011) and a history of miscarriages and abortions (Finnbogadóttir et al., 2014a; Nerum et al., 2010; Schei et al., 1991; Stenson et al., 2001) were identified as predicting factors for exposure to violence during pregnancy, but unplanned pregnancy may be sequelae of abuse for a variety of reasons.

A registered study from Finland revealed that 3% of all deaths linked to pregnancy, birth, abortion, or miscarriage during the period 1987 to 1994 were associated with an almost fourfold increased risk for all types of violent deaths. There was a more than fourfold risk for femicide after an induced abortion compared to women having miscarriages and those giving birth (Gissler & Hemminki, 1999).

Three Norwegian cross-sectional studies reported an increased risk of unplanned pregnancy among women with any history of violence (Drevin et al., 2020; Flaathen et al., 2021). A multi-country cross-sectional study reported corresponding findings for Iceland, Denmark, and Sweden (Lukasse et al., 2015).

Two Norwegian studies found a link between a significant increase in antenatal hospital admission and a history of violence (Grimstad et al., 1999b; Henriksen et al., 2013). Another two studies, based on the Norwegian Mother, Father, and Child Cohort Study (MoBa), reported an increased risk of complications during pregnancy for women with a history of violence, including hyperemesis, threatened preterm birth (Henriksen et al., 2013), and pre-eclampsia (Lukasse et al., 2010b). Reporting severe sexual violence had an almost twofold risk of being hospitalized with hyperemesis, threatening preterm birth, or being admitted more than once during pregnancy (Henriksen et al., 2013).

### Association Between A History of Violence and Physical Symptoms

Two MoBa-based studies indicated that women with a history of childhood abuse were more likely to report seven or more common pregnancy complaints than women not exposed to violence (Lukasse et al., 2009). Similarly, severe sexual violence experienced previously and recently was significantly associated with an almost sevenfold risk of suffering from more than eight pregnancy-related symptoms (Lukasse et al., 2012). Additionally, two case-control studies from Norway based on the same material indicated that abused pregnant women were less likely to suffer from uterus contractions, more likely to suffer from leg cramps (Grimstad et al., 1999b), and had significantly more health complaints compared to non-abused women (Grimstad & Schei, 1999). One cohort study from Sweden found that women exposed to violence during pregnancy had an increased risk of reporting more preceding ill health during pregnancy (Stenson et al., 2001). Additionally, women subjected to abuse more often had non-scheduled visits at the antenatal clinic (ANC), more discomfort, more self-reported genital infections (Grimstad & Schei, 1999), and more urinary tract infections during pregnancy (Stenson et al., 2001).

### Association Between A History of Violence and Psychological Consequences

A MoBa-based study found that both lifetime sexual and physical violence were significantly associated with binge eating disorder during pregnancy (Knoph Berg et al., 2011).

Lifetime exposure to violence was common among women seeking abortion. Exposure to violence was associated with low education, single marital status, smoking, and high alcohol consumption. Exposure to violence was associated with the occurrence of signs of posttraumatic stress disorder (PTSD) and symptoms of anxiety and depression.

A Swedish cross-sectional study with 1,514 women found that lifetime exposure to violence was common among women requesting termination of pregnancy under 12 weeks of gestation. Exposure to violence was associated with PTSD, anxiety, and depression in this group of women (Tinglöf et al., 2015). Similarly, a cross-sectional study in a culturally diverse population attending antenatal care in Norway showed that women with a history of IPV were almost twice as likely to report symptoms of antenatal depression compared to women with no history of violence (Melby et al., 2022). Likewise, three different Swedish studies, a cohort study of 1,939 pregnant women and two cross-sectional studies with 1,003 and 2,466 pregnant women, respectively (five articles), showed an association between symptoms of depression and DV (Finnbogadóttir, Baird et al., 2020; Finnbogadóttir & Dykes, 2016; Finnbogadóttir et al., 2014a, 2016; Finnbogadóttir & Mellgren, 2017; Wangel et al., 2016; Wikman et al., 2020). Additionally, Wangel et al. (2016) found that women who had experienced emotional, physical, or sexual abuse had a three- to twelvefold increased risk of experiencing symptoms of posttraumatic stress (Wangel et al., 2016).

In a recent cross-sectional study from Sweden involving 3,371 pregnant women, a 12% prevalence of high scores for depressive symptoms was observed. These high scores were significantly associated with an increased risk of all types of violence compared to women with lower depression symptom scores (Eikemo et al., 2023).

A low sense of coherence has been measured as significantly low with a more than threefold risk for women with a history of violence compared to women without a history of violence (Finnbogadóttir, Baird et al., 2020; Finnbogadóttir & Dykes, 2016).

In a MoBa-based study, mothers reporting adult abuse had an almost twofold increased risk of postpartum depression (PPD) (Sorbo et al., 2014). Involvement with known perpetrators, recent abuse, and reporting exposure to more types of abuse increased the likelihood of PPD (ibid). A longitudinal cohort study from Sweden with 2,466 pregnant women showed that having experienced IPV was associated with depression during the perinatal period (Wikman et al., 2020). A Swedish cross-sectional study found an association between anxiety, depression, sexual abuse, and emotional abuse (Hedin & Janson, 1999). Additionally, two cross-sectional studies, one from Sweden and a multicenter study including four Nordic countries (Denmark, Iceland, Norway, and Sweden), showed that pregnant women with a history of violence, regardless of its type, commonly experienced suffering at the time of the pregnancy as a consequence of the abuse (Finnbogadóttir & Mellgren, 2017; Lukasse et al., 2014).

A cross-sectional study from Iceland with 208 pregnant women visiting the emergency department or the high-risk prenatal care clinic showed that sexual abuse by a close family member, emotional abuse in the preceding 12 months, or current abuse predicted current psychological health symptoms (Svavarsdottir & Orlygsdottir, 2008). In a cross-sectional study from Norway with 1,812 pregnant women, women with a history of IPV were significantly more likely to report fear of their partner, after adjusting for confounding factors, at about a twofold risk compared to women without a history of IPV (Melby et al., 2022).

A longitudinal cohort study from Sweden with 2,466 pregnant women showed that having experienced IPV corresponded to an almost fourfold risk of early PPD onset respective late PPD onset, and more than a sevenfold risk of chronic depression (Wikman et al., 2020).

A cross-sectional MoBa-based study showed that women who reported a history of physical and sexual abuse in childhood were at an increased risk of severe worries about the baby’s health during pregnancy compared to women without this experience of abuse (Eide et al., 2010). Similarly, another study with the same cohort showed that nulliparous women who had been exposed to severe sexual violence had, compared to non-exposed women, an almost 50% increased risk of worries about the infant’s health and were not looking forward to the arrival of the infant (Henriksen et al., 2016). Women with lifetime exposure to sexual violence, regardless of the timing, were less likely to look forward to the infant’s arrival than non-exposed women (ibid). Additionally, a study from Norway showed that a history of abuse was associated with a negative birth experience (Henriksen et al., 2017). One study from Sweden estimating the prevalence of potentially traumatic events (PTE) where abuse was included, based on responses from 945 pregnant women, reported that most pregnant women had experienced at least one PTE and the prevalence of posttraumatic stress disorder was 4.1% (Persson et al., 2020).

Several studies from Norway showed an increased risk of fear of childbirth (FOC) for women with a history of abuse (Eberhard-Gran et al., 2008; Henriksen et al., 2016; Lukasse et al., 2014; Lukasse et al., 2011; Lukasse et al., 2010a; Størksen et al., 2015). A study based on data from Denmark, Iceland, Norway, and Sweden showed that a history of violence was significantly associated with an almost twofold higher risk of severe FOC (Lukasse, Schei et al., 2014). Women with a history of sexual abuse reported nearly five times the risk of extreme fear during labor compared to women without a history of abuse (Eberhard-Gran et al., 2008). Another multicenter study reported that being exposed to physical or sexual abuse in childhood increased FOC for both primi- and multiparous women. Still, the strongest association with severe FOC among multiparous women was an earlier negative birth experience (Lukasse et al., 2010a). Nulliparous women exposed to severe sexual violence had, compared to non-exposed women, a 50% increased risk of FOC (Henriksen et al., 2016). Additionally, a Danish multicenter cohort study showed that the experience of a severe history of violence was associated with an increased risk of FOC (Schroll et al., 2011).

A history of violence was also associated with reduced expectations of a positive childbirth experience and the wish to give birth by Cesarean section (CS) (Heimstad et al., 2006). A Swedish study estimating the prevalence of PTE, including experienced violence, with responses from 945 pregnant women, found the prevalence of FOC to be 28.8%, with 10.9 % of these reporting to have received support for FOC (Persson et al., 2020).

A MoBa-based study showed almost twice as many women using antiseizure medication (ASM) for untreated or treated epilepsy among women reporting any emotional, physical, or sexual abuse than among women without epilepsy. Having experienced abuse was associated with increased FOC in nulliparous women with ASM-untreated epilepsy but not in women with ASM-treated epilepsy (Vederhus et al., 2022).

### Association Between A History of Violence and Obstetric Outcomes

Several studies from Norway showed an association between a history of violence and an increased risk of experiencing induction of labor, epidural analgesia, instrumental and complicated delivery, or prolonged labor (Heimstad et al., 2006; Lukasse, et al., 2014; Lukasse et al., 2010b; Nerum et al., 2010, 2013). By contrast, one MoBa-based study found that severe sexual violence (rape) was associated with a reduced risk of episiotomy, and nulliparous women had a reduced risk of having anal sphincter injuries (Henriksen et al., 2014a).

A case-control study from Norway reported that women who had experienced rape before their first delivery had a thirteenfold increased risk for assisted vaginal delivery compared to controls and a significantly longer second stage of labor than the control group (Nerum et al., 2010). An Icelandic study also found an association between a history of violence and prolonged labor (Gisladottir et al., 2016). In addition, this study found an association between a history of violence and antepartum bleeding, an increased risk of maternal distress during labor, and emergency instrumental delivery (Gisladottir et al., 2016). A Danish cohort of obstetrically low-risk nulliparous women showed no association between a history of violence and labor dystocia at term (Finnbogadóttir et al., 2011).

Several Norwegian studies have reported an increased risk of giving birth by CS for women with a history of violence, both elective and emergency CS (Henriksen et al., 2014a; Lukasse et al., 2010b; Nerum et al., 2010, 2013). The multi-country study, including data from Denmark, Iceland, Norway, and Sweden, showed a twofold increased risk of elective CS among primiparous women with a history of abuse and an almost fourfold increased risk of CS without medical indication (Schei et al., 2014). Furthermore, a study from Sweden showed that a history of violence or a solely reported history of emotional abuse increased a woman’s risk of undergoing a CS, irrespective of whether it was an elective or an emergency CS (Finnbogadottir & Baird, 2020).

Research from Sweden indicates that infants born to mothers with a history of violence are more likely to be born prematurely (Finnbogadóttir, Baird et al., 2020), and women who experienced abuse during pregnancy had infants with a shorter gestational age (Stenson et al., 2001). By contrast, a study based on data from the national Norwegian MoBa cohort study showed no significant association between sexual violence and preterm birth (PTB), low birth weight (LBW), or small for gestational age (Henriksen et al., 2014b).

A study from Norway showed that newborns’ mean birth weight was significantly lower among women living in a physically abusive relationship compared to those not presently living in such a relationship (Schei et al., 1991). By contrast, two other studies from Norway showed no significant association between being subjected to abuse and giving birth to an infant with LBW (Grimstad et al., 1997, 1999b). Conversely, those who experienced a wide range of interpersonal conflict behavior and abuse showed marginal LBW (Grimstad et al., 1999b).

A study from Norway with a representative sample of 137 IPV mothers seeking help showed that the severity of physical IPV and injury from sexual IPV increased the risk of adverse consequences to the fetus (Vatnar & Bjørkly, 2011).

Data from the MoBa study showed that women exposed to child abuse were more likely to stop breastfeeding before 4 months than women without a history of childhood abuse (Sorbo et al., 2015). By contrast, a study from Sweden showed that women who had been exposed to DV during pregnancy and/or postpartum (all reported a history of violence) were just as likely to breastfeed as women who had not reported exposure to DV (Finnbogadottir & Thies-Lagergren, 2017).

### Interventions Used to Detect and Prevent Further Violence

A recent study from Denmark (and Spain) found that digital screening for IPV during ANC is feasible. However, providing a digital supportive intervention targeting pregnant women who screen positive for IPV is less viable, with only 21.4% of eligible women receiving the intervention (Andreasen et al., 2023).

A Norwegian RCT indicated that a digital storytelling intervention addressing IPV during pregnancy, health consequences, help-seeking strategies, and safety-promoting behaviors did not enhance participants’ quality of life, safety behavior utilization, or exposure to violence (Flaathen et al., 2022).

Summary of critical findings for the present scoping review are offered in Table 2.

**Table 2.** Summary of Critical Findings.

There is a knowledge gap in this research field in Finland.
Intervention studies to detect and prevent further violence are scarce.
The evidence for the association between the experience of violence and the risk of depression during the perinatal period is substantial.
A history of violence is associated with significantly more hospitalizations during pregnancy and impacts physical and psychological health. A history of violence impacts obstetric outcomes with risk for femicide, severe fear of childbirth, elective and emergency Cesarean section, and early cessation of breastfeeding. Several predictors for being exposed to violence during the perinatal period were identified.

## Discussion

This scoping review is the first to investigate comprehensive literature on the predictors for and consequences of a history of violence experienced by women in the Nordic countries during pregnancy and childbirth, the associated factors and predictors for perinatal violence exposure, and the interventions used to detect, address and prevent further violence. It covers evidence from the five Nordic countries over the past 32 years. However, we found a knowledge gap in this research field in Finland. The present review also identified scarce research, with only two studies assessing the impact of interventions targeting pregnant women exposed to violence. Additionally, the present review highlights the scarcity of longitudinal cohort and qualitative studies in the Nordic countries. However, the body of knowledge in this area has been growing since the beginning of the 2020s, and recent studies exhibit more robust designs with larger cohorts and longitudinal approaches.

Both the qualitative (Edin et al., 2010; Engnes et al., 2012; Finnbogadottir et al., 2014b; Kristinsdóttir & Halldórsdóttir, 2010) and quantitative studies (Grimstad et al., 1999a, 1999b; Lukasse et al., 2009; Lukasse et al., 2012) showed that women with a history of violence had more common complaints during their pregnancies. These findings correspond with a systematic review, showing that abused women often experience chronic health problems and injuries (O’Doherty et al., 2015). This is essential information for midwives and other healthcare professionals working in ANC, as common pregnancy complaints may indicate a history of violence and a requirement for extended care.

The present scoping review indicates that a history of violence is associated with significantly more hospitalizations during pregnancy (Grimstad et al., 1999b; Henriksen et al., 2013). This result is supported by studies from the USA (Cokkinides et al., 1999; El Kady et al., 2005; Weiss et al., 2002) and can be helpful information to midwives and other healthcare personnel working in hospitals. A Swedish qualitative study found that midwives in the in-hospital prenatal ward assumed that identifying pregnant women exposed to IPV should be the responsibility of their colleagues in ANC (Finnbogadóttir, Torkelsson et al., 2020). This attitude should be altered by providing continuous education and increasing midwives’ awareness.

This scoping review presents evidence from several studies, including those with cross-sectional and longitudinal cohort designs, indicating that a history of violence increases the risk of developing FOC (Eberhard-Gran et al., 2008; Heimstad et al., 2006; Henriksen et al., 2016; Lukasse, Schei et al., 2014; Lukasse et al., 2011; Lukasse et al., 2010a; Schroll et al., 2011; Størksen et al., 2015; Vederhus et al., 2022). Earlier research supports the finding that a history of sexual abuse may lead to FOC (Courtois & Courtois Riley, 1992; Simkin, 2009) and increases the risk for both planned and emergency CS (Courtois & Courtois Riley, 1992; Rachana et al., 2002). The association between a history of violence and FOC should increase awareness among midwives and other healthcare professionals. Reducing FOC is essential for the individual and may be a way to reduce the risk of CS among this group of pregnant women.

Several studies have found an increased risk for both elective and emergency CS among women with a history of violence, regardless of its type (Finnbogadóttir et al., 2020; Henriksen et al., 2014a; Lukasse et al., 2010b; Nerum et al., 2010, 2013; Schei et al., 2014). This finding highlights the importance of healthcare professionals being aware of this risk factor and ensuring support and care for this group of women.

The present scoping review found that pregnant women with a history of violence exhibit symptoms of depression (Edin et al., 2010; Eikemo et al., 2023; Engnes et al., 2012; Finnbogadóttir et al., 2020; Finnbogadóttir & Dykes, 2016; Finnbogadóttir et al., 2014a, 2016; Finnbogadottir et al., 2014b; Finnbogadóttir & Mellgren, 2017; Kristinsdóttir & Halldórsdóttir, 2010; Melby et al., 2022; Wangel et al., 2016; Wikman et al., 2020). Two earlier systematic reviews have supported the finding of an increased risk of depression among violence-exposed women during both the prenatal and postpartum periods (Alvarez-Segura et al., 2014; Howard et al., 2013). The evidence for the association between an experience of violence and the risk of depression continues to grow.

The mother’s well-being during pregnancy affects her emotional attachment to the fetus, and IPV has been independently found to be associated with a poor motherhood experience (Hooker et al., 2016). The motherhood experience, including the emotional attachment, is a critical factor for developing good parenting skills after birth and, therefore, for the child’s health (Foley & Hughes, 2018).

The present scoping review found no evidence indicating a link between being subjected to abuse and giving birth to an infant with LBW. By contrast, a systematic review and meta-analysis including 50 studies from populations in 17 countries across five continents showed that women subjected to IPV are predisposed to higher levels of stress-related hormones, which can lead to PTB and LBW (Donovan et al., 2016). Another systematic review with 15 studies from Asia, 12 from North America and Oceania, and 12 from Central and South America showed an association between IPV and PTB and LBW (Pastor-Moreno et al., 2020). The apparent contradiction between our findings and these reviews may be due to methodological differences. Another possible explanation might be that diversity across ethnicities, cultures, and the health care systems might influence the risk of LBW and PTB among women across continents subjected to violence. LBW is one of the leading causes of morbidity (Grigoriadis et al., 2013), and there should be increased awareness that pregnant women with a history of violence may be at higher risk of preterm birth.

Two studies examined the relationship between exposure to violence and breastfeeding (Finnbogadottir & Thies-Lagergren, 2017; Sorbo et al., 2015). A large population-based cohort study conducted in Norway revealed that women with a history of exposure to abuse were more likely to cease breastfeeding early compared to women without a history of violence (Sorbo et al., 2015). This finding was not supported by a cross-sectional study conducted in Sweden (Finnbogadottir & Thies-Lagergren, 2017); however, the contradictory results may be due to different study designs. A recent systematic review showed that exposure to IPV in any form and stage did not reduce breastfeeding initiation but was negatively associated with breastfeeding duration and early cessation of exclusive breastfeeding (Normann et al., 2020). Early breastfeeding cessation has short- and long-term consequences for mother and child, contributing to health inequality (Daoud et al., 2015; Walker et al., 2011). Research has shown that breastfeeding can increase maternal mental well-being (Victora et al., 2016) and strengthen the mother-child attachment (Weaver et al., 2018). Therefore, more knowledge about how women exposed to violence approach and experience breastfeeding and how we can best support this group of women in achieving a successful breastfeeding experience is essential.

### Strengths and Limitations

The first strength of this extensive scoping review is that it brought together evidence from the Nordic countries over the past 32 years and mapped the knowledge gaps in the literature. Furthermore, we applied a robust search strategy and included Finnish, Icelandic, Norwegian, Danish, and Swedish studies, gray literature from each country, and literature in all five Nordic languages. It is a strength that a librarian performed the literature search to reduce bias and that we conducted a recent update of the investigation. Another vital strength that helped mitigate bias was having different teams handle the review process when a reviewer had authored one or more articles. This ensured that the paper’s author was never solely responsible for data extraction, qualitative assessments, or synthesizing of results, further enhancing the fairness and objectivity of the review process. According to the scoping methodology used (O’Mallay, 2005; Peters et al., 2020), we included a wide range of papers, including gray literature, which we also consider a strength.

The study also has some limitations. Since we only included evidence from the Nordic countries, generalizability to other study populations may be limited. However, VAW is a global problem, and sociodemographic characteristics or gender inequality at the country level do not seem to explain the high prevalence of VAW in Nordic countries. Therefore, the rest of the world may benefit from the results of this scoping review. What may differ are sociocultural factors, healthcare systems, economy, and laws. Furthermore, we included all available studies on this topic to address the research questions, and the variation in quality among these studies may have influenced our comprehension of their findings.

Another limitation is that we could only assess causality to a limited extent due to several articles with cross-sectional designs. Finally, no further efforts were made to retrieve unpublished research, such as contacting authors. Despite these limitations, this scoping review contributes comprehensive knowledge in this vital field of study.

### Clinical Implications

To reduce the risk of both short- and long-term adverse health consequences for mother and child, it is crucial to identify pregnant women with a history of violence in early pregnancy and initiate appropriate care for this group of women (Table 3). A non-judgmental, open, and reassuring approach may help reduce stigma and fears about vulnerability (Forder et al., 2020). However, midwives and other healthcare professionals must have adequate resources as well as support and education to be able to identify and help pregnant women at risk of IPV (Johnsen et al., 2023). Indeed, all the Nordic countries have recommendations on how to address this delicate matter during the perinatal period (Helsedirektoratet, n.d.; Ministry of Health, 2012; Socialstyrelsen, 2014; Sundhedsstyrelsen, 2021; The Ministry of Social Affairs and Health, 2023). Appointments with the midwife or public health nurse at ANCs could be the key to opening a conversation about a woman’s life situation, including asking about violence.

**Table 3.** Summary of Implications for Practice, Policy, and Research.

Practice	Among professionals, awareness and continuous education are needed to change attitudes.
Policy	Consensus on screening for depression in early pregnancy in the Nordic countries would benefit the mother’s and child’s health outcomes.
Research	The body of research on the consequences of a history of violence on women’s pregnancy and childbirth is growing in all the Nordic countries, but in Finland, there is a knowledge gap. However, there is a need for more longitudinal cohort studies and intervention studies to address the consequences of violence and prevent further violence.

Particular attention should be paid to women with several hospitalizations and women who express complaints during pregnancy, as these may be indicators of a history of violence and a requirement for extended care. Moreover, it is essential to be aware that a history of violence may lead to FOC and, thereby, an increased risk of CS; therefore, it is relevant to evaluate strategies to reduce the FOC (Smith et al., 2019) among women with a history of violence. Screening for depressive symptoms in early pregnancy as well as postpartum needs to be considered as early intervention may increase the mother’s and child’s well-being also in a life perspective. Finally, special attention should be paid to women with a history of violence so that inequality in breastfeeding and, consequently, inequality in health in the short and long term is avoided.

## Conclusion

This scoping review offers comprehensive insights into the consequences of a history of violence among pregnant women in Nordic countries, as well as the associated factors and predictors for perinatal violence exposure. The findings reveal that violence-exposed pregnant women are at greater risk of, for example, common complaints and hospitalization during pregnancy, FOC, CS, breastfeeding difficulties, and mental health problems. This information is vital for healthcare professionals, especially midwives, to identify and support these women according to their needs.

Considering the various and severe consequences of a history of violence on women’s pregnancy and childbirth, this scoping review has shown the clinical effort that should be devoted to pregnant women we know have a history of violence. Many do not disclose the violence, so we want to promote the idea that all pregnant women should be supported as potential survivors of violence.

This review also underscores the existing gaps in the literature. Future research should focus on violence among pregnant women in Finland, Greenland, and the Faeroe Islands. In all Nordic countries, further development, implementation, and evaluation of effective interventions are needed to prevent violence and reduce its consequences. Furthermore, there is a need for more research on the well-being of this group of women during the postpartum period. Comprehensive research in this field can increase awareness and contribute to evidence-based care.

The results of this scoping review may hold implications for improving future antenatal care and, hopefully, fostering collaboration between the Nordic countries, both among researchers and in evaluating guidelines for addressing this critical and complex matter in the best possible way for the health of mothers and newborns.

## Supplemental Material

sj-docx-2-tva-10.1177_15248380241253044 – Supplemental material for The Consequences of A History of Violence on Women’s Pregnancy and Childbirth in the Nordic Countries: A Scoping Review Supplemental material, sj-docx-2-tva-10.1177_15248380241253044 for The Consequences of A History of Violence on Women’s Pregnancy and Childbirth in the Nordic Countries: A Scoping Review by Hafrún Rafnar Finnbogadóttir, Lena Henriksen, Hanne Kristine Hegaard, Sigridur Halldórsdóttir, Eija Paavilainen, Mirjam Lukasse and Lotte Broberg in Trauma, Violence, & Abuse

sj-docx-3-tva-10.1177_15248380241253044 – Supplemental material for The Consequences of A History of Violence on Women’s Pregnancy and Childbirth in the Nordic Countries: A Scoping Review Supplemental material, sj-docx-3-tva-10.1177_15248380241253044 for The Consequences of A History of Violence on Women’s Pregnancy and Childbirth in the Nordic Countries: A Scoping Review by Hafrún Rafnar Finnbogadóttir, Lena Henriksen, Hanne Kristine Hegaard, Sigridur Halldórsdóttir, Eija Paavilainen, Mirjam Lukasse and Lotte Broberg in Trauma, Violence, & Abuse

sj-docx-4-tva-10.1177_15248380241253044 – Supplemental material for The Consequences of A History of Violence on Women’s Pregnancy and Childbirth in the Nordic Countries: A Scoping Review Supplemental material, sj-docx-4-tva-10.1177_15248380241253044 for The Consequences of A History of Violence on Women’s Pregnancy and Childbirth in the Nordic Countries: A Scoping Review by Hafrún Rafnar Finnbogadóttir, Lena Henriksen, Hanne Kristine Hegaard, Sigridur Halldórsdóttir, Eija Paavilainen, Mirjam Lukasse and Lotte Broberg in Trauma, Violence, & Abuse

sj-docx-5-tva-10.1177_15248380241253044 – Supplemental material for The Consequences of A History of Violence on Women’s Pregnancy and Childbirth in the Nordic Countries: A Scoping Review Supplemental material, sj-docx-5-tva-10.1177_15248380241253044 for The Consequences of A History of Violence on Women’s Pregnancy and Childbirth in the Nordic Countries: A Scoping Review by Hafrún Rafnar Finnbogadóttir, Lena Henriksen, Hanne Kristine Hegaard, Sigridur Halldórsdóttir, Eija Paavilainen, Mirjam Lukasse and Lotte Broberg in Trauma, Violence, & Abuse

sj-docx-6-tva-10.1177_15248380241253044 – Supplemental material for The Consequences of A History of Violence on Women’s Pregnancy and Childbirth in the Nordic Countries: A Scoping Review Supplemental material, sj-docx-6-tva-10.1177_15248380241253044 for The Consequences of A History of Violence on Women’s Pregnancy and Childbirth in the Nordic Countries: A Scoping Review by Hafrún Rafnar Finnbogadóttir, Lena Henriksen, Hanne Kristine Hegaard, Sigridur Halldórsdóttir, Eija Paavilainen, Mirjam Lukasse and Lotte Broberg in Trauma, Violence, & Abuse

sj-rtf-1-tva-10.1177_15248380241253044 – Supplemental material for The Consequences of A History of Violence on Women’s Pregnancy and Childbirth in the Nordic Countries: A Scoping Review Supplemental material, sj-rtf-1-tva-10.1177_15248380241253044 for The Consequences of A History of Violence on Women’s Pregnancy and Childbirth in the Nordic Countries: A Scoping Review by Hafrún Rafnar Finnbogadóttir, Lena Henriksen, Hanne Kristine Hegaard, Sigridur Halldórsdóttir, Eija Paavilainen, Mirjam Lukasse and Lotte Broberg in Trauma, Violence, & Abuse
