# Supplementary material for: The Consequences of A History of Violence on Women’s Pregnancy and Childbirth in the Nordic Countries: A Scoping Review
Source: Trauma Violence Abuse. 2024 May 28;25(5):3555–70. doi: 10.1177/15248380241253044 (PMC11545221; doi:10.1177/15248380241253044)
Supplement: sj-docx-2-tva-10.1177_15248380241253044 – Supplemental material for The Consequences of A History of Violence on Women’s Pregnancy and Childbirth in the Nordic Countries: A Scoping Review [file sj-docx-2-tva-10.1177_15248380241253044.docx]

Appendix B. Data extraction form

| **The aim** was to explore the consequences of a history of violence on women’s pregnancy and childbirth in the Nordic countries and the interventions used to address these consequences and prevent further violence. | |
| --- | --- |
| ***Bibliographic information*** | |
| Study ID (EndNote number) |  |
| Reference |  |
| Extracted by |  |
| Date extracted |  |
| Checked by |  |
| Type of study |  |
| ***For research reports*** | |
| Country (or –ies) of research conduction |  |
| ***Aims, methods and results*** | |
| Study aims/objectives |  |
| Methodology |  |
| Methods |  |
| Data analysis |  |
| Intervention |  |
| Intervention outcome |  |
| ***Does the article present data relate to the following information?*** | |
| ***SURFACE STRUCTURE*** | |
| **Participants**  Women from the Nordic countries with history of violence during the perinatal period*.  *The perinatal period is defined as a period of time when you become pregnant and up to a year after giving a birth. |  |
| **Settings** |  |
| **Outcome**  **-**association of experienced violence  **-**consequence of experienced violence  **-** interventions used reduced or prevented the violence or negative consequences |  |
| **Conclusion** | |
|  | |
| ***Recommendations*** | |
| ***Decision*** | |
| Include ( ) | |
| Exclude ( ) | |
| ***Reasons for exclusion*** | |
