# Supplementary material for: The Consequences of A History of Violence on Women’s Pregnancy and Childbirth in the Nordic Countries: A Scoping Review
Source: Trauma Violence Abuse. 2024 May 28;25(5):3555–70. doi: 10.1177/15248380241253044 (PMC11545221; doi:10.1177/15248380241253044)
Supplement: sj-docx-3-tva-10.1177_15248380241253044 – Supplemental material for The Consequences of A History of Violence on Women’s Pregnancy and Childbirth in the Nordic Countries: A Scoping Review [file sj-docx-3-tva-10.1177_15248380241253044.docx]

**Table S1.** Overview of included studies with a Quantitative design (not cross-sectional design).

| **First author**  **(Year)**  **Country** | **Design** | **Setting, Recruitment, Population and Sample** | **Instruments and Exposure** | **Analysis** | **Main Outcome and Results** |
| --- | --- | --- | --- | --- | --- |
| Andreasen K.  (2023)  Denmark  ~~Spain~~ | A Nested cohort intervention study.  A feasibility study. | Pregnant women attending ANC were offered digital screening for IPV.  Only Danish data is presented here.  Data collection started in February 2021 and finished in October 2022.  17220 pregnant women were invited to complete the screening.  16,068 women (82.65%) completed the screening. | The intervention was a digital supportive intervention.  Women who screened positive were eligible to receive an intervention consisting of counselling (3-6 sessions) and a safety planning app during pregnancy.  Abuse Assessment Screen (AAS)  Women Abuse Screening Tool (WAST).  Index of Spouse abuse (ISA) | Descriptive statics  T-test | *Main outcome*: The outcomes of interest for the digital intervention were (1) reduction of the severity of IPV, (2) empowerment, (3) safety planning, (4) pre/postnatal depression, and (5) acceptability and (6) feasibility of the intervention.    A digital screening for IPV among pregnant women as part of antennal care is feasible and 8.53% were found exposed to IPV.  A digital supportive intervention targeting pregnant women who screen positive for IPV is less feasible, as only 21.4% of the women who were eligible for the intervention received it. |
| Finnbogadóttir, H.  (2011)  Denmark | A population-based multi-centre cohort study | The Danish Dystocia Study (DDS). Nine Antenatal Clinics at obstetric departments throughout Denmark between May 2004 - July 2005. N= 2652 pregnant obstetrically low-risk nulliparous women. | The short form of the Conflict Tactics Scale (CTS2S) | Pearson Chi-square test.  Univariate and multivariate regression analyses. | *Main outcome*: whether self-reported history of violence or experienced violence during pregnancy is associated with increased risk of labour dystocia in nulliparous women at term.  No association between history of violence and labour dystocia at term,  crude (OR 0.91, 95% CI 0.77-1.08), (OR 0.90, 95% CI 0.54-1.50), respectively.    Exposure to a ‘history of violence’ was more frequently reported by women who had a lower educational level (≤ 10 years) compared to women not exposed (p < 0.001). |
| Finnbogadóttir, H.  (2016a)  Sweden | A longitudinal cohort study | Recruitment ANC, total 19 midwife led clinics from multi-ethnic geographical areas in SV-Scania. Prospectively collected data between March 2012 -April 2014. A cohort of 1939 pregnant whereof 78.8 % or number of 1509 answered questionnaires, QI (early pregnancy) and QII (late pregnancy). | NorAQ  AAS (4 modified questions)  EPDS  AUDIT  SOC-13 | Descriptive statistics,  Pearson Chi-square test  Logistic and multiple regression analyses.  Adjusted for Single/living apart, EDS ≥13, Low SOC-score, Lack of sleep, financial distress,  Low educational status, Unintended pregnancy, and Age. | *Main outcome*: prevalence and incidence of domestic violence (DV) among pregnant women and their experience of a history of violence and to explore the association between DV during pregnancy and possible risk factors.  History of violence was the strongest single risk factor associated with DV during pregnancy (p < 0.001).  *Single/living apart* was associated with DV during pregnancy (AOR 8.4, 95 % CI 2.2–32.6).  *Several symptoms of depression* and *lack of sleep* was associated with DV respectively (AOR 3.8, 95 % CI 1.1–13.6) and (AOR 3.8, 95 % CI 1.1–12.9). |
| Finnbogadóttir, H.  (2016b)  Sweden | A longitudinal cohort study | 65 different Child-Welfare-Centres (CWC) from multi-ethnic geographical areas in SV-Scania.The third and the last questionnaire (Q3) was completed between1 to 1.5 years postpartum. Prospectively collected data between March 2012 - April 2015. A cohort of 1939 pregnant whereof 38.9% or number of 731 mothers. | NorAQ  AAS (4 modified questions)  EPDS  AUDIT  SOC-13 | Pearson Chi-square test  Logistic and multiple regression analyses.  Adjusted for Age Single/living apart, EPDS ≥ 13, Low, SOC-score, and Lack of sleep. | *Main outcome:* prevalence and incidence of DV during pregnancy and 1 to 1.5 years postpartum and their experience of a history of violence. Explore the association between DV postpartum and possible risk factors.  The strongest risk factor for DV reported was *a history of violence* whereby all the women (n = 23) exposed to DV postpartum also reported a history of violence (p < 0.001).  *Single/living apart* had a higher risk for DV postpartum (AOR 12.9, 95 % CI 4.5–37.1).  *Several symptoms of depression* and *a low score on the SOC-scale* was associated with a higher risk of DV (AOR 3.5, 95 % CI 1.2–10.4) and (AOR 3.0, 95 % CI 1.1–8.3), respectively. |
| Finnbogadóttir, H.  (2020)  Sweden | A longitudinal cohort study | Antenatal Care, total 19  midwife led clinics from multi-ethnic geographical areas in SV-Scania. Prospectively collected data, June 2012 - April 2014. Of 1939 pregnant women available dataset included birth records of 1694 mothers. | NorAQ  AAS (4 modified questions)  EDS  AUDIT  Birth records | Descriptive statistics Pearson Chi-square test  T-test  Bivariate logistic regression analyses  Adjusted for low socioeconomic status, cohabiting, employment, economy, and tobacco use on the rate of the two adverse pregnancy outcomes; premature childbirth and CS showed no potential cause and effect or significant association. | *Main outcome:* childbirth outcomes of women reporting a history of violence including domestic violence during pregnancy.  Women living apart from their partner, being unemployed, single, smoking and/or facing financial distress, were at increased risk of experiencing violence (p = 0.001). They also had significant low scores on the SOC-scale and had high EDS-scores ≥13 when compared to women without a history of violence (p = 0.001).  A history of violence increased the woman’s risk of undergoing a caesarean section (AOR 1.33, 95% CI 1.02–1.70).  A history of emotional abuse increased the risk of having a caesarean section irrespective of whether it was a planned or an emergency caesarean section (AOR 1.50, 95% CI 1.09–2.06).  Infants born to a mother who reported a history of violence, were at significant risk of being born premature < 37 weeks of gestation (p = 0,049) compared to mother reporting no history of violence. |
| Flaathen, E. M. E.  (2022)  Norway | A multicentre RCT | 19 MCHC with culturally diverse pregnant women located in southeastern Norway. Prospectively collected data between January 2018 - July 2019. Of 1818 pregnant women. 317 reported IPV, and total of 251 (79.2%) women completed the follow-up questionnaire. | WHOQOL-BREF  AAS  CASR-SF  The intervention video used digital storytelling and con- sisted of images, pictures, sound and a video, focusing on information about the definition and types of IPV, the cycle of abuse, IPV during pregnancy and health conse- quences, help-seeking strategies and safety-promoting behaviours. | Pearson Chi Square test,  Mann Whitney U, Logistic regression analysis | *Main outcome:* women’s perceived quality of life within the last two weeks.  The intervention did not improve the participants quality of life, use of safety behaviours or exposure to violence. |
| Gísladóttir, A.  (2014)  Iceland | Register based prospective cohort study | Linked data sources from the Rape Trauma Service  (RTS) and the Icelandic nationwide birth register (IBR) between March 1993 -December 2008. All subsequent pregnancies after attendance at the RTS were identified up to mid-April 2011. A population of 2556 (exposed 915; unexposed 1641 deliveries)  Icelandic women whereof 594 women (exposed group) subsequently became pregnant, resulting in a total of 925 deliveries. | Attended the RTS at least once- information from records.  The IBR contains information on parents and neonates  and International Classification of Diseases (ICD-10) codes. | Poisson log-linear models with robust error variance to obtain RR. | *Main outcome:* Characteristics and risk factors during pregnancy, including maternal smoking, body mass index, weight gain during pregnancy, illicit drug use.  Sexually assaulted women were ≤ 19 years  (RR: 2.13, 95% CI 1.49-3.04) and age group 20-24 years (RR: 1.46, 95% CI 1.22-1.74) and more often primiparous in subsequent pregnancy (RR: 1.31 95% CI 1.20-1.42) compared to women not sexually assaulted.  More likely to be *unemployed* (7.8% vs. 4.3%; ARR 2.42,95% CI 1.49–3.94), *not cohabiting* (45.6% vs. 14.2%; ARR 2.15, 95% CI 1.75–2.65), *smokers* (45.4% vs. 13.5%; ARR 2.68, 95% CI 2.25–3.20), and *use illicit drugs during pregnancy* (3.4% vs. 0.4%; ARR 6.27,95% CI 2.13–18.43). *Exposed primiparas were more likely to be obese* (15.5% vs. 12.3%; ARR 1.56, 95% CI 1.15–2.12) compared to women not sexually assaulted. |
| Gísladóttir, A.  (2016)  Iceland | A case control study | Linked data sources from the  RTS and the IBR. Women attending the RTS between 1993–2010 and delivered at least one singleton infant in Iceland through 2012. N = 1068 cohort. For each exposed woman's delivery, nine deliveries by women with no RTS attendance were randomly selected from the  IBR (n = 9126) matched on age, parity, and year and season of delivery. | RTS  IBR  ICD-10 codes. | Poisson log-linear models with  robust error variance to obtain RR. | *Main outcome:* Childbirth outcome  Compared to non-violence exposed women, exposed women presented with increased risks of maternal distress during labour and delivery (RR 1.68, 95% CI 1.01–2.79), prolonged first stage of labour (RR 1.40, 95% CI 1.03–1.88), antepartum bleeding (RR 1.95, 95% CI 1.22–3.07) and emergency instrumental delivery (RR 1.16, 95% CI 1.00–1.34).  No difference between the groups regarding the risk of elective caesarean section (RR 0.86, 95% CI 0.61–1.21), except for a reduced risk among those assaulted as teenagers (RR 0.56, 95% CI 0.34–0.93). |
| Gissler, M.  (1999)  Finland | Letter to editor, a  register study | National registers of deaths, births, induced abortions, and hospital discharges for the period 1987-94. Data linked to Medical Birth Register, the Abortion Register and the Hospital Discharge Register. N= 9192 women’s deaths, of which **3163** were violent, were individually linked to registers containing information on pregnancy. | National Registers of deaths  Medical Birth Register  Abortion Register  Hospital Discharge Register | Descriptive statistics  Logistic regression analysis | *Main outcome:* Deaths among 15-49-year-old women by preceding reproductive event.  Evidence of pregnancy, birth, abortion, or miscarriage was found in 281 cases or 3% of all deaths.  The risk for all types of violent deaths and homicide increased after an induced abortion respectively (AOR 3.73, 95% CI 1.95 - 7.14); (AOR 4.44, 95% CI 4.06 - 18.55) was increased, compared to women having miscarriages and giving birth. |
| Grimstad, H.  (1997)  Norway | A case-control study  (retrospective) | Women were recruited during an 18-months period in 1992–94 at the Department of Obstetrics, University Hospital of Trondheim.  86 women (cases) delivered of a low birthweight (< 2500 g) infant and 92 delivered of an infant with birthweight  **≥** 2500 g (controls). | In depth semi structured interviews  Conflict Tactile Scale (CTS) modified | Pearson Chi-square test  Mantel-Haenszel test  Multivariate analyses based on multiple Logistic regression models.  Adjusted for age, education, partners education, smoking status, pre-pregnancy weight | *Main outcome:* Low birthweight  There was no significant association between being subjected to abuse and giving birth to an infant with low birth weight (OR 1.37, 95% CI 0-63-2.99).  Abused women reported higher consumption of cigarettes in pregnancy compared with non-abused women, However, no difference between groups when adjusted for potential confounders, (AOR, 1.53, 95% CI 0.74-3.17). |
| Grimstad, H.  (1998)  Norway | A case-control study | Women were recruited during an 18-months period in 1992–1994 at the Department of Obstetrics, University Hospital of Trondheim. A retrospective study.  N= 178, Case group (n=86)  Control group (n=92) | Semi structured interviews Birth records  CTS (modified) | Pearson Chi-square test  Multiple logistic regression  Adjusted for educational level | *Main outcome:* consumption of cigarettes and alcohol during pregnancy among women with a history of sexual abuse or a history of physical abuse.  A history of abuse was associated with daily smoking (AOR 2.29, 95% CI 1.08–4.85) and with alcohol consumption in pregnancy (AOR 2.57, 95% CI 1.22–5.39). |
| Grimstad, H.  (1999a)  Norway | A case-control study | Women were recruited during an 18-months period in 1992–94 at the Department of Obstetrics, University Hospital of Trondheim. A retrospective study.  N= 178  86 women (cases) delivered of a low birthweight (< 2500 g) infant and 92 delivered of an infant with birthweight  **≥** 2500 g (controls). | CTS modified  Trait-Anxiety Scale  (T-Anxiety) | Pearson Chi-square test  Student's t-test  ANOVA  Pearson's correlation  A logistic regression models.  Multivariate analyses | *Main outcome:* to assess the relationships among anxiety, history of abuse and low birth weight.  A high level of anxiety and/or a history of abuse was not associated with low birth weight (OR 1.17, 95% CI 0.60 - 2.30).  A history of child sexual abuse was associated with higher mean health complaint scores (3.0, SD 1.0) compared to no history of child sexual abuse (2.5, SD 0.7, p=0.02). However, no significant difference when comparing mean health complaint score among abused vs. non-abused. |
| Grimstad, H.  (1999b)  Norway | A case-control study | Women were recruited during an 18-months period in 1992–94 at the Department of Obstetrics, University Hospital of Trondheim. A retrospective study.  84 women (cases) delivered of a low birthweight (< 2500 g) infant and 90 delivered of an infant with birthweight  **≥** 2500 g (controls). | In depth semi structured interviews  CTS modified  Birth records | Pearson Chi-square test  Student’s *t* tests  Mann-Whitney U 2-tailed tests Multivariate analyses, Logistic regression, and Multiple linear regression models.  Adjusted for Intrauterine growth restriction (IUGR) | *Main outcome:* The association between being exposed to abuse and Birth weight.  No association between abuse and LBW was found (OR 0.75, 95% CI 0.42–1.37).  Experienced interpersonal conflict behavior.  and abuse in a wide sense and lower birth weight with 261 g (*p* < 0.045).  Abused women were younger than non-abused, and more often not married or cohabitating.  Cases who reported abuse in pregnancy were  more frequently delivered by a caesarean section compared with non-abused women (*p =* 0.05).  There was a significant relation between any abuse during the relationship and admission to hospital during pregnancy (AOR 8.55, 95% CI 1.01–72.61).  Abused were less likely to suffer from intrauterine contractions than non-abused and more likely to suffer from leg cramps or aching legs or feet in pregnancy. |
| Grimstad, H.  (1999c)  Norway | A case-control study | Women were recruited during an 18-months period in 1992–94 at the Department of Obstetrics, University Hospital of Trondheim. A retrospective study. 82 women (cases) with birth of a low-birth-weight infant  (2500g) and 91 women (controls) with birth of a normal birth weight infant | In depth semi structured interviews  CTS modified  Birth records | Pearson Chi-square test  Fisher’s exact tests  Student’s *t*-tests  Mann Whitney *U* tests  Multivariate analyses were based on multiple logistic and multiple linear regression models. | *Main outcome:* The association between being exposed to abuse and Birth weight.  No association were seen between a history of child sexual abuse and LBW (OR 1.03, 95% CI .44 –2.40).  Abused women reported more health complaints compared to non-abused: Heartburn/Regurgitation,  (*p* = 0.03), Pelvic Joint Syndrome (*p* < 0.01), Back Pain,  (*p* = 0.04) Compared to non-abused, abused women had more often a non-scheduled visit at the antenatal clinic (AOR 3.34, 95% CI 1.06 10.46), a higher self-reported genital infection with Candida and Gardnerella (*p =* 0.01) and a higher mean score of discomfort (*p = 0*.01). |
| Henriksen, L.  (2013)  Norway | A population-based cohort study | The Norwegian MoBa is a prospective population-based pregnancy cohort study conducted by the Norwegian Institute of Public Health. Pregnant women who self-reported at 17- and 30-weeks’ gestation during pregnancy through postal questionnaires.  Years 1999-2008.  N = 78 660 pregnant women. | History of sexual violence was reported at three levels:  Mild: pressured to sexual acts. Moderate: forced with violence.  Severe: raped.  The comparison group did not report sexual violence. | Pearson Chi-square test  Fishers exact test  Logistic regression analyses  Adjusted for age, parity, BMI | *Main outcome:* whether a history of sexual violence was associated, with an increase in antenatal hospitalizations.  A history of sexual violence was associated with significantly more hospitalizations during pregnancy, 6.6% (n = 625) for mild, 8.7 % (n = 193) for moderate and 12.5 % (n = 351) for severe compared to 5.8 percent for no sexual violence (p< 0.001).  Reporting severe sexual violence had a risk for being hospitalized with hyperemesis or threatening preterm birth (AOR 1.9, 95% CI 1.4–2.5), and (AOR1.9, 95% CI 1.3–2.7) respectively.  Severe sexual violence was associated with being admitted more than once during pregnancy (AOR 1.9, 95% CI 1.3–2.7). |
| Henriksen, L.  (2014a)  Norway | A populations-based cohort study | MoBa is a prospective population-based pregnancy cohort study conducted by the Norwegian Institute of Public Health. Pregnant women who self-reported at 17- and 30-weeks’ gestation during pregnancy through postal questionnaires. Recruitment between 1999 - 2008.  N =74 059 pregnant women | History of sexual violence was reported at three levels:  Mild: pressured to sexual acts. Moderate: forced with violence.  Severe: raped.  The comparison group did not report sexual violence.  Data from the MoBa study were linked with data from the Medical Birth Registry of Norway (MBRN)  Mental distress measured by The Hopkins Symptom  Checklist, which accounted for five items (SCL-5) | A multivariable logistic regression analysis  Adjusted for age, education, body mass index, smoking, diabetes, pre-eclampsia, induced birth, dystocia, epidural, macrosomia, mental distress, previous caesarean section . | *Main outcome:* Mode of delivery and selected maternal birth outcomes.  Women with a history of sexual violence were significantly younger, unemployed, and less likely to be living with a partner compared to women without a history of sexual violence. More frequently reported smoking and alcohol consumption during early pregnancy, having a body mass index ≥30 kg/m2, and mental distress.  Severe sexual violence (rape) was associated with elective caesarean section (AOR 1.56, 95% CI 1.18– 2.05) for nulliparous women and (AOR 1.37, 95% CI 1.06–1.76) for multiparous women, with risk of induction, with an (AOR1.22, 95% CI 1.04–1.42) and (AOR 1.37, 95% CI 1.15–1.63) for nulliparous and multiparous women, respectively, and with a reduced risk of episiotomy and nulliparous had a reduced risk of sphincter tear.  Exposed to moderate sexual violence had a higher risk of emergency caesarean section, (AOR 1.31, 95% CI 1.07–1.60) and (AOR 1.41 95% CI 1.08–1.84) for nulliparous and multiparous women, respectively. |
| Henriksen, L.  (2014b)  Norway | A populations-based cohort study | Women were recruited to MoBa while attending routine ultrasound examinations between 1999 - 2008. Pregnant women who self-reported at 17- and 30-weeks’ gestation during pregnancy through postal questionnaires.  N = 76 870 pregnant women | History of sexual violence was reported at three levels:  Mild: pressured to sexual acts. Moderate: forced with violence.  Severe: raped.  The comparison group did not report sexual violence.  Neonatal outcomes were retrieved from the MBRN. | A linear and logistic regression analysis | *Main outcome:* not a specific  There was no significant association between sexual violence and pre-term birth (PTB), low birthweight (LBW) or small for gestations age (SGA) in the adjusted analysis.  Moderate and severe violence had a small but significant effect on gestational age (2 days).  Women reporting moderate or severe sexual violence had a significantly reduced gestational length 277.6 days (β −2.02, CI 95% −3.39 to −0.67) and 277.5 days (β −1.92, CI 95% −3.22 to −0.62), respectively, when the birth was provider-initiated compared to non-abused. (279.7 days)  Women exposed to sexual violence in this study reported more of the sociodemographic and behavioural factors associated with PTB, LBW, and SGA compared with non-abused women. |
| Henriksen, L.  (2016)  Norway | A populations-based cohort study | Women were recruited to the MoBa while attending routine ultrasound examinations between 1999 - 2008. Pregnant women who self-reported at 17- and 30-weeks’ gestation during pregnancy through postal questionnaires.  N = 78 660 pregnant women. | History of sexual violence was reported at three levels:  Mild: pressured to sexual acts. Moderate: forced with violence.  Severe: raped.  The comparison group did not report sexual violence.  MBRN  SCL-25 | A multivariable logistic regression analysis  Adjusted for Maternal age, parity, socio-economic status and civil status, mental disease | *Main outcome:* FOC (but also other defined as main)  18.4% reported a history of sexual violence and 0.9% were exposed to sexual violence within the last 12 months, including during the current pregnancy.  Nulliparous women who were exposed to severe sexual violence had compared to non-exposed.  FOC (AOR 1.5, 95% CI 1.3–1.7), thoughts about pain relief (AOR 1.3, 95% CI 1.1–1.5), worries about the infant’s health (AOR 1.4, 95% CI 1.2–1.6) and were not looking forward to the arrival of the infant (AOR 0.9, 95% CI 0.7–1.2). |
| Lukasse, M.  (2011)  Norway | A longitudinal cohort study | Data from MoBa conducted by the Norwegian Institute of Public Health. Fifty maternity units in Norway, data collection between, 1999–2006.  N = 4 876 Norwegian women who participated in the MoBa study during their first and second pregnancy. | NorAQ  (a modified version)  Data from the MoBa study were linked with data from MBRN | Logistic regression  Adjusted for mode of first delivery and experience of first birth | *Main outcome:*  Associations between childhood abuse and women’s FOC and preference for caesarean section during second pregnancy.  Compared to women without a history of childhood abuse, childhood-abused women more frequently reported FOC (23% vs. 15%, p < 0.001) (AOR 1.53, 95% CI 1.24–1.90) and a wish for caesarean section (6.4% vs. 4.0%, p < 0.002) during second pregnancy (AOR 1.57, 95% CI 1.09–2.27). |
| Lukasse, M.  (2012)  Norway | A population-based national cohort study | Women were recruited to MoBa while attending routine ultrasound examinations at  17 and 32 weeks of gestation between 1999 - 2008.  N = 78 660 Norwegian pregnant women. | NorAQ (a modified version)  Data from the MoBa study were linked with data from MBRN | Pearson’s Chi-square test  Logistic regression  Adjusted for age, child physical violence, adult physical violence, child emotional abuse and adult emotional abuse. | *Main outcome:* the association between sexual violence and the reporting of physical symptoms during pregnancy.  Compared to women not reporting sexual violence, women reporting sexual violence had a higher risk of suffering from ≥8 pregnancy-related symptoms (AOR 1.49, 95% CI 1.41–1.58) for mild sexual violence, (AOR 1.66, 95% CI 1.50–1.84) for moderate and (AOR 1.78, 95% CI 1.62–1.95) for severe. Severe sexual violence (previously and recently) had association with suffering from ≥8 pregnancy-related symptoms (AOR 6.70, 95% CI 2.34–19.14). |
| Nerum, H.  (2010)  Norway | A case-control study | The women who had been raped as adults > 16 years were recruited from a cohort of 808 pregnant women referred to counselling by the mental health team at the antenatal clinic at University Hospital of North Norway, between 2000 and 2007.  The women were referred in subsequent pregnancies for various psychosocial burdens, such as history of anxiety and  depression, eating or sleep disturbances, post-traumatic  stress disorder, fear of birth or a previous traumatic birth experience.  Cases = 50 women raped as adults (> 16 years) were compared with 150 controls. | The electronic journal system PARTUS® | Pearson’s Chi-square test  Mann–Whitney U  A multinomial logistic regression  Adjusted for age, marital status, employment status, BMI, previous abortions, obstetric risk level, oxytocin augmentation, epidural analgesia, and birthweight | *Main outcome:* Caesarean section, operative vaginal delivery, and duration of labour.  Women who had been raped at their first delivery had an increased risk for caesarean section (AOR 15.7, 95% CI 5.0–49.1) and for assisted vaginal delivery (AOR 13.1, 95% CI 4.9–34.5) when compared with controls. The group of women who had been raped had a longer second stage of labour than the control group (120 versus 55 minutes, P < 0.01). They were more often *single mothers*, *unemployed* and *smokers*, and had *a higher body mass index* and *more previous pregnancy terminations and miscarriages* than the control group. |
| Nerum, H.  (2013)  Norway | A case-control study | The women who had been raped as adults > 16 years were recruited from a cohort of 808 pregnant women referred to counselling by the mental health team at the antenatal clinic at University Hospital of North Norway, between 2000 and 2007.  The women were referred in subsequent pregnancies for various psychosocial burdens, such as history of anxiety and depression, eating or sleep disturbances, post-traumatic  stress disorder, fear of birth or a previous traumatic birth experience.  373 primiparas and 185 were subjected to Child sexual abuse (CSA), 47 to RA and 141 controls without a history of abuse. | The electronic journal system PARTUS® | Pearson’s Chi-square test  Kruskal–Wallis test  Multinominal regression analysis.  Adjusted for: not specified | *Main outcome:* Vaginal births, delivery by caesarean section, operative vaginal delivery, and duration of labour.  As compared with controls, the RA group showed a significantly higher risk for caesarean section (AOR 9.9, 95% CI 3.4–29.4) and operative vaginal delivery (AOR 12.2, 95% CI 4.4–33.7). There were no significant differences between the CSA and the control group. The RA group displayed significantly longer duration of labour in all phases as compared with the control and CSA groups. |
| Schei, B.  (1991)  Norway | A Case control study | Women aged 20-49 year living in Trondheim in Norge.  The Women’s Aid Refuge Center and through a randomly selected group of women. Twenty-eight women were recruited from the Emergency Clinic, where they were treated for their inflicted injuries. Between December 1987-January 1988.  Cases are 66 women aged 20-49 living in a physically abusive relationship.  Controls are 114 women randomly selected and not presently living in such a relationship were interviewed postpartum.  306 pregnancies included in the analysis. | Structured interviews | Pearson’s Chi-square test  T-tests  ANOVA  A linear regression model  Adjusted for education, primiparity and history of addiction reduced the difference in mean birth weight to 175 g. | *Main outcome:*  Adverse outcome of pregnancy.  Exposure to violence in pregnancy was associated with low education.  The index cohort had experienced more miscarriages than women in the control  cohort, 16.1% vs. 9.6% (p < 0.05).  The mean birthweight was significantly lower among births in the index cohort compared in the control cohort (3329 vs. 3482 g; *p* < 0.05)  After adjustment borderline significance. |
| Schroll, A. M.  (2011)  Denmark | A Cohort study | The DDS. Nine Antenatal Clinics at obstetric departments throughout Denmark. Data collection between May 2004 to July 2005.  N = 2638 obstetrically low-risk nulliparous women. | W-DEQ  Delivery Fear Scale (DFS) CTS2S  Birth records | T-test.  Spearman’s rank correlation, r.  Mann–Whitney U-test. | *Main outcome:*  To estimate the prevalence of self-reported lifetime violence and to assess whether women exposed to any physical violence or sexual violence (SEV) had a higher risk of having FOC.  Experience of severe violence (SEV) was associated with an increased risk of severe FOC after delivery, OR 1.5 (95% CI: 1.02–2.27).  Women who experienced violence were *younger,* more often *had an unplanned pregnancy,* *lived more frequently without a partner,* and *more often had never commenced or interrupted an education.* They had an *unhealthier lifestyle* with regards to *use of tobacco, alcohol and medicine during pregnancy.* |
| Sørbo, M. F.  (2014)  Norway | A populations-based Cohort study | Women were recruited to MoBa while attending routine ultrasound examinations at  17–18 weeks of gestation between 1999 - 2008.  N = 53 065 pregnant women and women 6 months postpartum, n = > 49 000 | EPDS  NorAQ (a modified version)  AAS | Logistic regression  Adjusted for age, education, depression prior to pregnancy, social support, and civil status | *Main outcome:* the association between adult abuse and Postpartum depression (PPD).  Women reporting adult abuse had increased risk of PPD (AOR 1.8, 95% CI 1.7-1.9) PPD was higher for women with any recent adult abuse (AOR 2.6, 95% CI 2.4-2.9) and higher for women with any adult abuse, but not recent (AOR 1.5, 95% CI 1.5-1.7).  Women reporting three types of abuse; emotional, physical, and sexual abuse, had a increased risk of PPD (AOR 2.2, 95% CI 1.9-2.6). |
| Sørbo , M. F.  (2015)  Norway | A populations-based Cohort study | Women were recruited to MoBa while attending routine ultrasound examinations at  17–18 weeks of gestation between 1999 - 2006. Prospectively collected data.  Mothers with singleton pregnancy who had responded to three questionnaires (weeks 18 and 30 in pregnancy, and 6 months postpartum) and had answered minimum one of the abuse questions in week 30.  N=53 934 mothers participated in MoBa. | NorAQ  AAS  EDS  The Norwegian Medical Birth  Registry (NMBR). | Descriptive statistics  Pearson’s Chi-square test  Binary logistic regression  Adjusted for  maternal age, education, civil status  and child abuse.  In addition, evaluation of the change in estimates  when including intermediate variables: smoking,  alcohol intake, parity, preterm delivery, social support,  mode of delivery, BMI, preterm delivery, and depression  prior to pregnancy. | *Main outcome:* whether exposure to past and recent emotional, sexual, or physical abuse was associated with early breastfeeding cessation (before 4 months postpartum), secondly whether a potential association differed for known and unknown perpetrators.  Any breast-feeding cessation for women exposed to any child abuse was (OR 1.41, 95% CI 1.32 to 1.50) compared with no abuse in childhood.  Women reporting emotional abuse only (AOR 1.28, 95% CI 1.18 -1.39), emotional and physical abuse (AOR 1.39, 95% CI 1.18-1.62), emotional and sexual abuse (AOR 1.27, 95% CI 1.02 -1.58) or those reporting all three types of abuse, that is, emotional, sexual and physical (AOR 1.47, 95% CI 1.23-1.76) were more likely to stop any breast feeding before 4 months than women without abuse. |
| Stensson K.  (2001)  Sweden | A cohort study | All women registered between Sept 1997 and February 1998 at all ANC clinics in Uppsala and planning to give birth in Uppsala were consecutively recruited for participation.  N = 1038 women | CTS  Index of Spouse Abuse (lSA) AAS  Medical birth register | Mann-Whitney U test  Fisher’s exact test | *Main outcome:*  none  Women abused during pregnancy reported more preceding ill-health (p = 0.03) and more elective abortions (p = 0.001) than non-abused women, urinary tract infections  (p = 0.02) during pregnancy and/or gave birth  to an infant of shorter gestational age  (p = 0.04). They were more likely not to be cohabiting (p= 0.01). |
| Størksen, H.T.  (2015)  Norway | A population-based cohort study | Routine antenatal care at Akershus University Hospital (Ultrasound screening at 18 weeks) and follow-up of those participating during pregnancy postnatally.  Self-administered questionnaires at 17- and 32-weeks’ gestation and 8 weeks postpartum.  N= 4662 women in the total cohort from the Akershus Birth Cohort. n =1984 women answered all three questionnaires and comprised the baseline sample for this study and n =1789 was analysed. | W-DEQ  AAS (postpartum)  EPDS  Oslo Social Support Scale  The Hopkins Symptom Check List (SCL-25)  A numeric rating scale (NRS)  A maternity and birth records | Pearson’s Chi-square test  A logistic regression analysis | *Main outcome:*  to investigate (a) the demographic and psychosocial characteristics associated with fear of childbirth and (b) the relative importance of such fear on both caesarean delivery preference and delivery  by elective CS.  Women with a history of sexual abuse measured postpartum were more likely to have a fear of childbirth measured during pregnancy coerced and forced sexual abuse (OR 1.7, 95% CI 1.1–2.7), (OR 2.0, 95% CI 1.0–3.9) respectively. |
| Wikman, A.  (2020)  Sweden | A population‐based longitudinal cohort study | N= 2466 pregnant women Recruited to the BASIC study at the first Ultrasound in gestations week 17. | EDS-10  Five trajectory groups of depressive symptom onset were created using the EPDS ≥13 (pregnancy) or ≥ 12 points (postpartum)  Lifetime Incidence of Traumatic Experiences (LITE)  Birth records | Multinomial logistic regressions analysis | *Main outcome:*  Maternal perinatal depression (PND)  Having experience IPV is associated with depression in all five trajectories.  Healthy (60.6%) reference  Pregnancy depression (8.5%), (OR 4.7, 95% CI 3.0–7.3)  Early postpartum onset (10.9%), (OR 3.6, 95% CI 2.4–5.5)  Late postpartum onset (5.4%), (OR 3.7, 95% CI 2.1–6.3)  Chronic depression (14.6%), (OR 7.4, 95% CI 5.2–10.5)  An *unplanned pregnancy* and severe fear of delivery was associated with *pregnancy depression*, *early postpartum onset,* and *chronic depression*, but not *late postpartum onset*. Being *a single parent*, having a *partner who did not help with the infant*, and *not breastfeeding* were characteristics associated with all PND trajectories. |

Abbreviation list: AAS (Abuse Assessment Screen); ANC (Antenatal Care); ANOVA (One-way analysis of variance); AOR (Adjusted Odds Ratio); ARR (Adjusted relative risk); AUDIT (Alcohol Use Disorder; Identification Test); BED (Binge eating disorder); BIDENS (Belgium, Iceland, Denmark, Estonia, Norway, Sweden); BMI (body mass index); CS (caesarean section); CTS2S (Conflict Tactics Scale 2 short form); CWC (Child-Welfare-Centres); DDS (the Danish Dystocia Study); DFS (Delivery Fear Scale); DV (domestic violence); ED (the emergency department); EPDS (Edinburgh Postnatal Depression Scale); FOC (Fear of Childbirth); HADS (Hospital Anxiety and Depression Scale); HRPCC (the high-risk prenatal care clinic); IBR (the Icelandic nationwide birth register); ICD-10 (International Classification of Diseases); IPV (intimate partner violence); ISA (Index of Spouse abuse); IUGR (Intrauterine growth restriction); LBW (low birthweight); LEC-5 (the Life Events Checklist); LITE (Lifetime Incidence Traumatic Experiences); MBRN (the Medical Birth Registry of Norway); MCHC (maternal and child health centres); MoBa (Norwegian Mother, Father and Child Cohort study); NorAQ (the Norvold Abuse Questionnaire); NRS (a numeric rating scale); OR (Odds Ratio): PCL (the PTSD Checklist); PMWI (Psychological Maltreatment of Women Inventory); PND (maternal perinatal depression); PPD (postpartum depression); PTB (pre-term birth); PTE (potentially traumatic events); PTS (post-traumatic stress); PTSD (post-traumatic stress disorder); RCT (a randomized controlled study); RR (relative risk); RTS (Rape Trauma Service); SCL-5 (the Hopkins Symptom Checklist, five items); SEV (severe violence/sexual violence); SGA (small for gestations age); SOC13 (Sense of Coherence Scale); SQ-PTSD (Screen Questionnaire post-traumatic stress disorder); STAI (the State-Trait Anxiety Inventory); SVAW (the Severity of Violence Against Women Scale); T-Anxiety (Trait-Anxiety Scale); TOP (termination of pregnancy); TSC-33 (the Trauma Symptom Checklist); VAS (Visual Analogue Scale); W-DEQ (Wijma Delivery Expectancy/Experience Questionnaire) WAST (Women Abuse Screening Tool)
