# Supplementary material for: The Consequences of A History of Violence on Women’s Pregnancy and Childbirth in the Nordic Countries: A Scoping Review
Source: Trauma Violence Abuse. 2024 May 28;25(5):3555–70. doi: 10.1177/15248380241253044 (PMC11545221; doi:10.1177/15248380241253044)
Supplement: sj-docx-4-tva-10.1177_15248380241253044 – Supplemental material for The Consequences of A History of Violence on Women’s Pregnancy and Childbirth in the Nordic Countries: A Scoping Review [file sj-docx-4-tva-10.1177_15248380241253044.docx]

**Table S2.** Overview of included studies with a Cross-sectional design.

| **First author**  **Year**  **Country** | **Setting, Recruitment, Population and Sample** | **Instruments and Exposure** | **Analysis** | **Main Outcome and Results** |
| --- | --- | --- | --- | --- |
| Drevin, J.  **(**2020)  Norway | Norwegian Mother and Child Cohort study (MoBa). Routine antenatal care of 50 of Norway’s 52 maternity units. Prospectively collected data approximately at gestational week 18 between 1999-2008. Total 112 745 pregnancies and n=76 197 pregnant women included in present study. | Non-validated questions about exposure to emotional, physical, and sexual abuse. | Poisson regressions relative risk (RR).  Adjusted relative risk (ARR).  Adjusted for education, age, and country of childbirth. | *Main outcome*: An unplanned pregnancy (yes/no).  Exposure to childhood emotional (ARR 1.14, 95% CI 1.10–1.19), physical (ARR 1.11, 95% CI 1.04–1.18) and sexual (ARR 1.20, 95% CI 1.14–1.27) abuse increased the risk of having an unplanned pregnancy compared to women without history of abuse.  Exposure to all three categories of abuse compared with none has an increased risk of having an unplanned pregnancy (RR 1.46 95% CI 1.35 to 1.58). |
| Eberhard-Gran, M.  (2008)  Norway | All women residing in two communities, prospectively collected data between 1998-1999. Women received a questionnaire 6 week´s post-partum at Child Health Clinics. N=414 postnatal women. | Abuse assessment screen (AAS).  Fear of Childbirth (FOC)  No fear/some fear/extreme fear during labour. | Logistic regression  Odds Ratio (OR)  Multivariate logistic regression analyses.  Adjusted Odds Ratio (AOR).  Adjusted for depression in pregnancy, duration of labour and mode of delivery | *Main outcome:* Occurrence of extreme fear  during labour and its association with sexual abuse in adult life.  Women with a history of sexual abuse had (OR 1.9, 95% CI 1.0–3.7) for reporting some fear during labour and or reporting extreme fear (OR 3.7, 95% CI 1.1–12.9) compared to women without a history of abuse.    The adjusted odds ratio for extreme fear during labour was (AOR 4.9, 95% CI 1.2–19.1). |
| Eide, J.  (2010)  Norway | Data from the Norwegian MoBa. Routine antenatal care, ultrasound at hospital approximately at 18 weeks of gestation, between 1999–2006.  N=60 775 pregnant women | The Norvold Abuse Questionnaire (NorAQ) - a modified, non-validated version.  Questions about abuse and worries about the infant´s health were answered in the 17th and 30th weeks of gestation. | Multivariate logistic regression analyses  Adjusted for education, age, marital status, parity, relationship with partner, antenatal care (good/distrust), pelvic pain, Anxiety/depression, and self-efficacy. | *Main outcome:*  Strong worries about the baby’s health in the 30th week of gestation among women with experience of childhood abuse.  Women reported being exposed to physical and/or sexual childhood abuse were at increased risk of strong worries about the baby’s health compared to women who had not been similarly exposed (AOR 1.62, 95% CI 1.26–2.08), (AOR 1.30, 95% CI 1.03–1.64; AOR 2.10, 95% CI 1.68–2.62) respectively. |
| Eikemo, R  (2023)  Sweden | Antenatal Care (ANC), total of 35 midwifery-led clinics in the capital town of Stockholm.  Prospectively collected data between October and December 2020. N = 3371 pregnant women. | AAS  Edinburgh Postnatal Depression Scale (EPDS) | Descriptive statistics  Bivariate logistic regression analysis. | *Main outcome*: prevalence of physical, psychological, and sexual intimate partner violence among pregnant women and to investigate potential associations between exposure and sociodemographic characteristics and health.  2.1% of the women reported exposure to intimate partner violence during pregnancy.  Twelve percent of the women had a high score on possible *depression symptoms* (EPDS ≥ 13) and had a significantly increased risk of exposure to all categories of IPV (physical, psychological, and/or sexual IPV and physical and psychological IPV separately) when compared to women with a lower score on *depression symptoms* (EPDS score *<* 13).  Women who were “living with a partner and child/children” had an increased risk of exposure to IPV (OR 2.47, 95% CI 1.41–4.36). “Living alone” or “living with parents or other adults” (OR 11.8, 95% CI4.93–27.4) respectively (OR 9.73, 95% CI 1.37–43.3).  Women *born in countries outside Europe* reported a significantly greater risk of exposure to IPV (OR 2.05, 95% CI 1.09–3.73) than women born in Europe and almost quadrupled risk of being exposed to physical IPV (OR 3.55, 95% CI 1.19–10.4).  *Unemployed* women had a significantly increased risk of exposure to IPV compared to employed women (OR 2.11, 95% CI 1.16–3.72). |
| Finnbogadóttir, H.  (2014a)  Sweden | Antenatal Care (ANC), total 19 midwife led clinics from multi-ethnic geographical areas in SV-Scania.  Prospectively collected data between March 2012-September 2013.  N = 1939 pregnant women. | NorAQ  AAS (4 modified questions)  Edinburgh Postnatal Depression Scale (EPDS)  Alcohol Use Disorder Identification Test (AUDIT)  Sense of Coherence Scale  (SOC-13) | Descriptive statistics,  Pearson Chi-square test.  Bivariate and Multiple regression analyses.  Relative Risk (RR)  Adjusted for low socio-economic status, miscarriage/  abortion, single/living apart, lack of sleep, unemployment, age, and parity. | *Main outcome*: prevalence of domestic violence among pregnant women and associations with risk factors.  A history of violence was the strongest single risk factor associated with DV during pregnancy (p < 0.001).    Several symptoms of depression were associated with risk of DV during pregnancy (AOR 7.0, 95% CI 1.9-26.3).    Women born outside Nordic countries i.e.. were overrepresented to be exposed to DV during pregnancy (RR, 2.4).    DV were significant more reported by *unemployed* (OR 5.1, 95% CI 1.7-15.9), those who were S*ingle or living* (OR 6.9, 95% CI 2.4-19.7), and having *a history of miscarriages and abortions* (OR 7.6, 95% CI 2.8-20.6). |
| Finnbogadóttir, H.  (2017a)  Sweden | ANC, total 19 midwife led clinics from multi-ethnic geographical areas in SV-Scania. Prospectively collected data between March 2012-September 2013. A cohort of 1939 women in early pregnancy. Dataset of 761 (39.5%) women who had history of violence was analyzed. | NorAQ  AAS (4 modified questions)  EPDS  AUDIT  SOC-13 | Descriptive statistics, Pearson Chi-square test  T-test | *Main outcome:* the degree of self-reported suffering following violent incidents and the prevalence of police reporting as well as other help-seeking behaviour among women in early pregnancy with history of violence.  More than four of five women (80.5%) having a history of emotional abuse (n = 374), more than half (52.4%) having history of physical abuse (n = 561), and almost three of four (70.6%) who experienced sexual abuse (n = 302) reported in the early second trimester of their pregnancy that they still suffered from their experience.  Women with a history of violence had significantly lower SOC-13-scores (p = 0.001) and a significantly higher EPDS-score (p = 0.001) than women without a history of violence. |
| Finnbogadóttir, H.  (2017b)  Sweden | 65 different Child-Welfare-Centres (CWC) from multi-ethnic geographical areas in SV-Scania. Questionnaire III (Q-III) completed. Prospectively collected data between March 2012 - April 2015. A cohort of 1939  women in early pregnancy.  Dataset of 731 (38.9%) mothers was analysed. | NorAQ  EPDS  SOC-13 | Descriptive statistics Pearson Chi-square test | *Main outcome:* How DV during pregnancy and postpartum influence breastfeeding prevalence and duration.  DV did not influence breastfeeding prevalence or duration.  Women who were exposed to DV during pregnancy and/or postpartum (all reported a history of violence) were just as likely to breastfeed as women who had not reported exposure to DV. |
| Flaathen, E. M. E.  (2021)  Norway | 19 maternal and child health centres (MCHC) with culturally diverse pregnant women located in southeaster Norway. Prospectively collected data between January 2018 - July 2019. N = 1778. | AAS  Composite Abuse Scale R-SF | Pearson Chi-square test  Mann–Whitney U test  Binary logistic regression analysis  Adjusted for age, civil status, education, occupation, family income, ethnicity, parity, tobacco use, and alcohol use. | *Main outcome:* Pregnancy intendedness  Women who reported some experience of IPV in their lifetime were more likely to experience an unintended pregnancy than women who had not experienced IPV (AOR: 1.74, 95% CI 1.23–2.47). |
| Hedin, L.W.  (1999)  Sweden | Three antenatal clinics in different socio-economic areas in Gothenburg. Prospectively collected data between February 1996 -March 1997.  N = 207 Swedish born pregnant women married or cohabitant to Swedish born men. | Structured interviews, face to face.  The Severity of Violence Against Women Scale (SVAW)  STAI  Psychological Maltreatment of Women Inventory (PMWI)  The Trauma Symptom Checklist (TSC-33) | Pearson Chi-square test  One way ANOVA  Correlations | *Main outcome:* none  A high correlation was found for anxiety depression, post-sexual abuse and PMWI.  Abused women were more often unemployed than non-abused. |
| Heimstad, R.  (2006)  Norway | A routine ultrasound scan at 18 weeks of gestation at St. Olav’s University Hospital, Trondheim between June 2001 and August 2002.  Questionnaire booklets were sent to N = 2680 women at 18 weeks of gestation, of whom 1452 women (54%) responded. | Wijma Delivery Expectancy/ Experience Questionnaire (W-DEQ)  The State-Trait Anxiety Inventory (STAI)  Pregnancy outcome information was recorded. | Pearson Chi-square test  Logistic regression analysis  Student’s t -tests  Fisher’s exact tests | *Main outcome:* the prevalence of fear of childbirth.  Women who reported being exposed to physical abuse in childhood had a higher W-DEQ score (71, SD 31) than the non-abused (61, SD 23, p </0.01).  Sexual abuse in childhood influenced the W-DEQ-score similarly (69, SD 27 versus 61, SD 23, (p =/0.05).  A history of physical or sexual abuse in adult life did not influence the W-DEQ score.  Risk estimates for complicated current delivery related to physical abuse in childhood (OR 2.3 95% CI 1.5-4.4) and sexual abuse in childhood (OR 2.5 95% CI 1.4-3.9). 57% and 54%, respectively, had uncomplicated vaginal deliveries at term, compared to 75% among those who did not report abuse (p < 0.001). |
| Henriksen, L.  (2017)  Norway | A study based on self-reported data from the Norwegian cohort of the BIDENS study.  N= 1352 multiparous women. | NorAQ  EDS-5  W-DEQ | Pearson Chi-square test.  A multivariable logistic regression analysis.  Adjusted for age, parity, education, cohabiting, economic hardship, history of abuse, social support, fear of birth, depression, previous vaginal birth. | *Main outcome:* If a negative birth experience was associated with fear of birth and a history of abuse.  A negative birth experience was associated with a history of abuse (AOR 1.34, 95% CI 1.01–1.79). |
| Knoph Berg, C.  (2011)  Norway | Data from MoBa study.  Recruitment at routine antenatal care filled out a questionnaire at about 19 weeks of gestation.  N = 45 644 pregnant women | Binge eating disorder (BED) defined as at least weekly episodes of binge eating.  Physical abuse measured by single question: “have you ever in your adult life been slapped, hit, kicked, or bothered in any way physically? With answering option: no never, yes during pregnancy, yes 6 months before pregnancy, and yes earlier in life. | A Poisson regression approach estimates of RR  Adjusted for age, education, and income | *Main outcome:* A negative experience was associated with fear of birth and a history of abuse.  Both lifetime sexual and physical abuse was associated with incidence of BED in pregnancy  (ARR 1.57, 95% CI 1.35–1.81) respectively (ARR 1.68, 95% CI 1.44–1.97).  No association between lifetime sexual and physical abuse were seen with BED continuation or BED remission in pregnancy (ARR 1.15, 95% CI 1.00 –1.33) respectively (ARR 1.11, 95% CI 0.95–1.30). |
| Lukasse, M.  (2009)  Norway | Data from MoBa study.  Recruitment at routine antenatal care, questionnaire at 16-20 weeks of gestation. Prospectively collected data between 2000 - 2006. N = 61 865 datasets of pregnancies and comprised women who returned both the baseline and the third questionnaire during pregnancy (at approximately 16–20 and 30–34 weeks of gestation).  Data of 5653 pregnancies of women who participated more than once  (i.e., only the first pregnancy was included), leaving a total of 55 776 women for analyses. | NorAQ (a modified version)  Data from the MoBa study were linked with data from MBRN | Regression analyses  caesarean section  Univariate logistic regression  One-way analysis of variance (ANOVA)  Adjusted for: age, civil status, education, occupation, parity, use of alcohol or smoking during pregnancy, and body mass index (BMI). | *Main outcome:* Not a specific  Heartburn, constipation, backache, headache, nausea and vomiting, candidiasis, urinary incontinence, urinary tract infection, pruritus gravidarum, pelvic girdle relaxation, leukorrhea, tiredness, leg cramps, and oedema in pregnancy were associated with reported childhood abuse.  Women reporting childhood abuse were more likely to report seven or more common complaints in pregnancy (AOR 1.7, 95% CI 1.6–1.9) for emotional abuse (AOR 2.5, 95% CI 2.0–3.1) for combined physical and sexual abuse and (AOR 3.5, 95% CI 3.0–4.0) for all three kinds of abuse. |
| Lukasse, M.  (2010a)  Norway | Multi-centre study: five Norwegian cities. The number of deliveries ranged between 1300 - 3400 births per year. Recruitment between January  2008 - March 2009. N = 2365 pregnant women. | NorAQ (a modified version)  W-DEQ  EDS-5  Fear of childbirth (FOC): a range of six options, from agreeing completely to disagreeing totally. Agreeing completely with the statement “I am really dread- ing giving birth” were defined as fearing labor | Student *t* tests  One-way ANOVA  Chi-square test  Fisher’s exact test  Adjusted for; age, education, civil status, planned pregnancy, adult abuse, and depressive symptoms. | *Main outcome:* the association between self-reported history of childhood abuse and fear of childbirth.  The mean WDE-Q score for women with a history of childhood abuse was 60.96 (SD: 24.52) compared with 56.57 (SD: 21.84) for women not reporting childhood abuse (p < 0.001).  Women with a history of childhood abuse reported severe and extreme fear of labour significantly more often than those without a history of childhood abuse, 18% versus 10% (*p* = 0.001), 5.7 versus 3.2 % (*p* = 0.008) respectively.  Emotional abuse showed the overall strongest association with severe fear of childbirth for both primiparas (OR 3.66, 95% CI 2.21–6.06) and multiparas (OR 1.75, 95% CI 1.04–2.94) compared to women with no childhood abuse.  The association between a history of childhood abuse and severe fear of childbirth remained significant after adjustment for confounding factors for primiparas (AOR 2.00, 95% CI 1.30–3.08).  The strongest association with severe fear of childbirth among multiparas was a negative birth experience (AOR 5.50, 95% CI 3.77–8.01). |
| Lukasse, M.  (2010b)  Norway | Women were recruited to MoBa while attending routine ultrasound examinations at  17–18 weeks of gestation between 1999 - 2008.  Participants in the study gave birth between January 2000 - December 2006. N = 26 923 primiparous women with singleton pregnancies at term.  The cohort MoBa Of all women, 18.8% (5060) had experienced any childhood abuse, 14.3% (3856) reported emotional abuse, 5.2% (1413) reported physical abuse and 6.4% (1730) reported sexual abuse. | NorAQ (a modified version)  Data from the MoBa study were linked with data from MBRN | Pearson’s Chi-square test  A multivariate logistic regression models.  Adjusted for: age, education, BMI, adult abuse, macrosomia and pre-eclampsia | *Main outcome:* an association between self-reported exposure to sexual, physical, and emotional childhood abuse and birth by caesarean section.  Women reporting childhood abuse were significantly *younger*, had completed *less education*, were *less likely to be employed* and *fewer lived with their partner* compared with women reporting no childhood abuse (*p* < 0.001). Obesity was significantly more frequent among women reporting childhood abuse (*p* < 0.001).  Compared to women not exposed to abuse during childhood, women abused in childhood more often preferred birth by caesarean section (2.7% vs. 2.0%, *p* = 0.001), had a higher prevalence of pre-eclampsia (5% vs. 4.2%, *p* = 0.013), more often had their birth induced (15.2% vs. 13.7% (*p* = 0.006), and more often received epidural analgesia (42.1% vs. 38.5% *p* < 0.001).  There was a significant increase in caesarean sections during labour when all forms of childhood abuse were included (AOR 1.16, 95% CI 1.03–1.30) compared to non-abused. |
| Lukasse, M.  (2014a)  ~~Belgium~~  Iceland  Denmark  ~~Estonia~~  Norway  Sweden | A European multicentre study, BIDENS where most of the women were from the Nordic countries Iceland, Denmark, Norway, and Sweden. Recruitment between March 2008-August 2010.  N = 6 870 pregnant women  Iceland (n = 585)  Denmark (n = 1252)  Norway (n = 2351)  Sweden (n = 958) | NorAQ  FOC was assessed by the  W-DEQ version A  EDS (short version) | Logistic binary regression analyses.  Adjusted for age, gestational age, education, and parity. | *Main outcome:*  to compare the prevalence, content, and associated factors of FOC in six European countries.  Any lifetime abuse was associated with severe FOC (AOR 1.81, 95% CI 1.55–2.11). |
| Lukasse, M.  (2014b)  ~~Belgium~~  Iceland  Denmark  ~~Estonia~~  Norway  Sweden | A European multicentre study, BIDENS where most of the women were from the Nordic countries Iceland, Denmark, Norway, and Sweden. Prospectively collected data between March 2008-August 2010.  N = 7200 and of them  5338 pregnant women attending routine antenatal care in the Nordic countries.  Iceland (n = 602)  Denmark (n = 1290)  Norway (n = 2424)  Sweden (n = 1022) | NorAQ  A Visual Analogue Scale VAS. | Pearson’s chi-squared test.  Kruskal-Wallis test. | *Main outcome:*  to investigate the prevalence of a history of abuse among women attending routine antenatal care and current suffering from reported abuse.  Any lifetime abuse reported in  *Iceland* 35.5 %, current severe suffering, emotional 23.5%, physical 15.5%, sexual 15.0%.  *Denmark* 33.6% current severe suffering emotional 15.1%, physical 8.0%, sexual 8.3%.  *Norway* 37.1%, current severe suffering emotional 11.5%, physical 4.0 %, sexual 8.4%.  *Sweden* 30.2%, current severe suffering emotional 18.1%, physical 14.4 %, sexual 13.9%. |
| Lukasse, M.  (2015)  ~~Belgium~~  Iceland  Denmark  ~~Estonia~~  Norway  Sweden | A European multicentre study, BIDENS where most of the women were from the Nordic countries, Iceland, Denmark, Norway and Sweden. Prospectively collected data between March 2008 - August 2010  N = 7102 and of them  5289 pregnant women attending routine antenatal care in the Nordic countries.  Iceland (n = 598)  Denmark (n = 1276)  Norway (n = 2403)  Sweden (n = 1012) | NorAQ | Pearson’s Chi-square test  Binary logistic regression analysis  Adjusted for age, civil status, education,  gestational age and economic hardship | *Main outcome:*  The prevalence of unintended pregnancy in six European countries and to investigate the association with a history of physical, sexual, and emotional abuse.  The prevalence of an unintended pregnancy among women not reporting any abuse was 15.8 % compared to 24.5 % among those reporting any lifetime abuse. The prevalence of an unintended pregnancy among women reporting any lifetime abuse was 24.5 %, and 38.5 % among women reporting recent abuse.  Women with a history of any lifetime abuse had significantly higher odds of unintended pregnancy, also after adjusting for confounding factors for any lifetime abuse (AOR 1.4, 95 % CI 1.23–1.60) and for recent abuse (AOR 2.03, 95 % CI 1.54–2.68). However, the adjusted association between unintended pregnancy and any lifetime abuse was only significant for Norway and Sweden. |
| Melby T.C.  (2022)  Norway | At routine antenatal care setting at 19 MCHC with culturally diverse from Safe Pregnancy study between January 2018 and July 2019. N = 5426 pregnant women whereof n =1812 included in present study. | Validated instruments measured quality of life, physical and mental health, depressive symptoms and IPV.  EPDS-5  AAS (modified version) | Pearson’s Chi-square test  Logistic regression,  Adjusted for background variables: age, civil status, joint family income, mother tongue, unintended pregnancy, tobacco use, alcohol, and alcohol partner | *Main outcome:*  the prevalence of antenatal depression and the association between symptoms of antenatal depression and physical, emotional, and sexual abuse in a culturally diverse population attending antenatal care.  More than one in ten (14%) women reported symptoms of antenatal depression.  Women with a history of IPV were significantly more likely to report symptoms of antenatal depression, after adjusting for confounding factors (AOR1.96, 95% CI 1.35–2.83). Similar results were found regarding fear of partner (AOR 2.02, 95% CI 1.32–3.07), emotional IPV (AOR 1.83, 95% CI 1.23–2.74), physical IPV (AOR 2.35, 95% CI 1.37–4.04) and sexual IPV (AOR 2.09, 95% CI 1.03–4.23). |
| Persson, A.  (2020)  Sweden | Recruitment of 1057 women in late pregnancy attending lectures in preparation for childbirth at hospitals in Stockholm.  945 pregnant with response rate of 81.7 % answered anonymously. Data on pregnant women giving birth within Region Stockholm was extracted (n = 40 344) | The Life Events Checklist forDSM-5  Extended Self-Report (LEC-5).  WDEQ  The PTSD Checklist (PCL) | Prevalence  Logistic regression  Mean scores and SD | *Main outcome:* Estimate prevalence of potentially traumatic events (PTEs), FOC, and support for it as well as posttraumatic stress disorder (PTSD) among pregnant women.  Most pregnant women, 78.5 percent (95% CI 75.6–81.3) reported having experienced at least one PTE.  FOC was found among 28.8 percent (95% CI 25.7–32.0) of pregnant women, while only 10.9 percent (95% CI 10.5–11.2) received support for FOC.  The prevalence of current PTSD was 4.1 percent (95% CI 2.8–5.8). |
| Ryding, E.L.  (2015)  ~~Belgium~~  Iceland  Denmark  ~~Estonia~~  Norway  Sweden | A European multicentre study, BIDENS where most of the women were from the Nordic countries, Iceland, Denmark, Norway and Sweden. Prospectively collected data between March 2008 - August 2010.  6870 pregnant women attending routine antenatal care.  Iceland (n = 585)  Denmark (n = 1252)  Norway (n = 2351  Sweden (n = 958)  Total 6422 in the analysis.  3189 primipara  3233 multiparas | NorAQ  WDEQ  EDS-5 | Pearson’s Chi-square test  Logistic regression  Adjusted for age, education, country, smoking in pregnancy, EDS score ≥ 7, and birthweight for primiparous women. Additionally, adjusted for previous caesarean for multiparous women. | *Main outcome:* Fear of childbirth and Caesarean section (CS)  Among primiparous women, those reporting severe fear of childbirth were more likely to give birth by elective caesarean, (OR, 1.66, 95% CI 1.05–2.61).  Among multiparous women, severe fear of childbirth increased the risk of elective caesarean (OR 1.87, 95% CI 1.30–2.69).  Reporting lack of positive anticipation, one of six dimensions of fear of childbirth, was most strongly associated with elective caesarean (OR 2.02, 95% CI 1.52–2.68).  Indications for caesarean were more likely to be reported as “nonmedical” among those with severe fear of childbirth; 16.7 % versus 4.6 % in primiparous women, and 31.7% versus 17.5% in multiparous women. |
| Schei, B.  (2014)  ~~Belgium~~  Iceland  Denmark  ~~Estonia~~  Norway  Sweden | A European multicentre study, BIDENS where most of the women were from the Nordic countries, Iceland, Denmark, Norway, and Sweden. Prospectively collected data at routine antenatal care between March 2008 and August 2010.  N = 6724 pregnant women  Iceland: n= 585  Denmark: n= 1268  Norway: n= 2234  Sweden: n= 944  3308 Primiparous  3416 Multiparous | NorAQ  W-DEQ  EPDS-5  Birth records | Binary and multinomial regression analysis  Adjusted for age, twin pregnancy, gestational age at birth, and country of residence. | *Main outcome:*  An operative delivery  categorized as an elective CS or an operative vaginal birth, or an emergency CS.  Among primiparous women, sexual abuse as an adult (>18 years) increased the risk of an elective CS (AOR 2.12, 95% CI 1.28–3.49).  Adult sexual abuse increased the odds of a CS without a medical indication, (OR 3.74, 95% CI 1.24–11.24).  Primiparous women expressing current suffering from the reported adult sexual abuse had the highest risk for an elective CS, (AOR 4.07, 95% CI 1.46–11.3).  Physical abuse had an increased risk for emergency CS, (AOR 1.51, 95% CI 1.05–2.19). |
| Svavarsdóttir, E. K.  (2008)  Iceland | The emergency department (ED) and the high-risk prenatal care clinic (HRPCC) the Landspitali University Hospital. Data collection between January - May 2006 at ED and between March - October 2006 at HRPCC.  N = 208 women in their first trimester of pregnancy 101 visiting the emergency department (ED) and 107 visiting the HRPCC. | Women Abuse Screening Tool (WAST)  The health-symptoms scale  The Evaluation  Interview Frame for Nurses and Midwives | Descriptive statistics  T-test  Stepwise regression  analysis | *Main outcome:* the effects of physical, sexual, or emotional abuse on physical and psychological health  Regression analysis: sexual abuse by a close family member, emotional abuse in the preceding 12 months, and current abuse, predicted current psychological health symptoms (adjusted R 2=0.442; p<0.000). For women in the HRPCC, results indicated that physical abuse, sexual abuse by a close family member, and a current abusive relationship significantly predicted the current psychological health (adjusted R2=0.320; p<0.000).  Those who reported having been physically abused within a close relationship, reported significantly better psychological health compared to women who did not report previous sexual or physical abuse. However, previous, or current abuse did not predict current physical health symptoms. In addition, the interaction of prior sexual abuse and the WAST total score for both groups of women did not predict psychological symptoms. |
| Tinglöf, S.  (2015)  Sweden | Outpatient clinics at the Obstetrics and Gynecology departments of six public hospitals in Sweden. A multicentre study with prospective collection of data. Baseline data from the PADIA study was used.  N = 1514 women requesting termination of pregnancy (TOP) with a gestational length less than 12 pregnancy weeks. | Screen Questionnaire-Post Traumatic Stress Disorder (SQ-PTSD)  Hospital Anxiety and Depression Scale (HADS) | Descriptive statistics  Pearson’s Chi-square test  A logistic regression analyses  Adjustment for age, marital status, education, smoking and alcohol | *Main outcome:* The association of exposure to violence with PTSD, anxiety, and depression among women with unintended pregnancies.  Exposure to violence was associated with *low education*, *single marital status*, *smoking,* *alcohol consumption,*  Women exposed to physical violence had a higher risk for PTSD (AOR 30.2, 95% CI 13.1–69.50), symptoms of depression (AOR 1.87, 95% CI 1.48-2.16), and anxiety (AOR 2.32, 95% CI 1.87-2.92). For women exposed to sexual violence symptoms of depression (AOR 2.34, 95% CI 1.69 -3.23), and anxiety (AOR 3.01, 95% CI 2.12-4.33). |
| Vatnar, S. K. B.  (2011)  Norway | A representative sample of 137 IPV help-seeking mothers was interviewed.  The sample was recruited from 10 shelters (*n* = 73), 5 police districts (*n* =41), and  6 family counselling agencies (*n =* 43) in Norway.  According to official records, shelters, police, and family counselling agencies cover about 85% of IPV help-seeking women in Norway. | Structured Sociodemographic Questionnaire.  Semi structured Intimate Parental Violence Questionnaire.  CTS2  British Crime Survey | Friedman test  Pearson’s Chi-square test  Univariate and multivariate logistic regression | *Main outcome:* mothers’ perception of the effects of intimate partner violence (IPV) during pregnancy and children’s exposure to IPV.  Severity of physical IPV (OR 1.80, 95% CI 1.13–2.88) and injury from sexual IPV (OR 2.36 95% CI1.00–5.62), increased the risk of negative consequences to the foetus.  25.5% of the children had been exposed before they were 1 year old, 40% before the age of 5.  Twenty-seven percent of the women who had  mutual children with the perpetrator reported that the children also had been targets of his physical violence and that 37.1% had been targets of psychological IPV. None reported sexual abuse of shared children.  Corresponding figures for perpetrator abuse of a child whom the woman had with another father was 12.4% for physical victimization, 21.2% for psychological victimization,  and 4.1% for sexual abuse. |
| Vederhus J.  (2022)  Norway | Register data from the Norwegian Mother, Father, and Child Cohort Study 1999–2008.  93 949 women without epilepsy  295 women with ASM-treated epilepsy, 318 women with ASM-untreated epilepsy identified through MBRN | NorAQ  Childbirth expectations questionnaire (30 weeks of gestation) | Logistic regression analyses where the association between epilepsy and experiences of abuse adjusted for maternal age and socioeconomic status. | *Main outcome:* prevalence of self-reported emotional, physical, and sexual abuse and childbirth expectations in pregnant women with epilepsy and association between having experienced abuse and childbirth expectations, particularly FOC.  Women with epilepsy more often reported experiences of abuse than women without epilepsy.  A total of 115 women (47%) with ASM-treated  epilepsy and 132 women (47%) with ASM-untreated epilepsy reported any emotional, physical, or sexual abuse, compared to 25 100 women (32%) without epilepsy (AOR 1.8, 95% CI 1.4–2.3) and (AOR 1.8, 95% CI 1.4–2.2), respectively.  More women with epilepsy (11%) also reported that they had been raped compared to women without epilepsy (4 %). Having experienced abuse was associated with increased fear of childbirth in nulliparous women with ASM-untreated epilepsy, but not in women with ASM-treated epilepsy. |
| Wangel, A. M.  (2016)  Sweden | Recruitment at ANC.  N=1003 pregnant women, 78.6% were native and 21.4% were non-native Swedish-speakers. | NorAQ  EPDS-5 Scale-5  Symptoms of Posttraumatic Stress (PTS) | Pearson’s Chi-square test  Fisher’s exact test  Multivariate logistic regression analyses  Adjusted for increasing age in years, education, marital status and experiencing financial distress. | *Main outcome:* PTS and symptoms of depression  Emotional and sexual abuse were significantly associated with symptoms of depression for both natives (AOR 1.92, 95% CI 1.07–3.43) respectively (AOR 1.97, 95% CI 1.12–3.45) and non-natives (AOR 5.09, 95% CI 2.19–11.85) respectively (AOR 2.91, 95% CI 1.03–8.22) compared to women not reporting any abuse.  Physical abuse was significantly associated with symptoms of depression for non-natives only (AOR 3.94 95% CI 1.63–9.49) compared to women not reporting any abuse.  Emotional, physical, and sexual abuse were significantly associated with symptoms of PTS for both native (AOR 3.42, 95% CI 1.88–6.24), (AOR 2.96, 95% CI 1.55–5.65), (AOR, 3.74, 95% CI 2.09–6.71) and non-native Swedish-speakers (AOR 8.26, 95% CI 3.30–20.71), (AOR, 12.14, 95% CI 4.56–32.36) (AOR 3.69, 95% CI 1.20–11.34) compared to women not reporting any abuse. |

Abbreviation list: AAS (abuse assessment screen); ANC (Antenatal Care); ANOVA (One-way analysis of variance); AOR (Adjusted Odds Ratio); ARR (Adjusted relative risk); AUDIT (Alcohol Use Disorder Identification Test); BED (binge eating disorder); BIDENS (Belgium, Iceland, Denmark, Estonia, Norway, Sweden); BMI (body mass index); CS (caesarean section); CWC (Child-Welfare-Centres); DV (domestic violence); ED (the emergency department); EPDS (Edinburgh Postnatal Depression Scale); FOC (Fear of Childbirth); HADS (Hospital Anxiety and Depression Scale); HRPCC (the high-risk prenatal care clinic); IPV (intimate partner violence); LEC-5 (the Life Events Checklist) ; MBRN (the Medical Birth Registry of Norway); MCHC (maternal and child health centres); MoBa (Norwegian Mother and Child Cohort study); NorAQ (the Norvold Abuse Questionnaire); OR (Odds Ratio); PCL (the PTSD checklist); PMWI (Psychological Maltreatment of Women Inventory); PTE (potentially traumatic events); PTS (post-traumatic stress); PTSD (post-traumatic stress disorder); RR (relative risk); SOC-13 (Sense of Coherence Scale); SQ-PTSD (Screen Questionnaire-post-traumatic stress disorder); STAI (the State-Trait Anxiety Inventory); SVAW (the Severity of Violence Against Women Scale); TOP (termination of pregnancy); TSC-33 (the Trauma Symptom Checklist); VAS (a Visual Analogue Scale); W-DEQ (Wijma Delivery Expectancy/Experience Questionnaire); WAST (Women Abuse Screening Tool)
