# Supplementary material for: The Consequences of A History of Violence on Women’s Pregnancy and Childbirth in the Nordic Countries: A Scoping Review
Source: Trauma Violence Abuse. 2024 May 28;25(5):3555–70. doi: 10.1177/15248380241253044 (PMC11545221; doi:10.1177/15248380241253044)
Supplement: sj-docx-5-tva-10.1177_15248380241253044 – Supplemental material for The Consequences of A History of Violence on Women’s Pregnancy and Childbirth in the Nordic Countries: A Scoping Review [file sj-docx-5-tva-10.1177_15248380241253044.docx]

**Table S3.** Overview of included studies with a Qualitative design.

| **First Author/Year/Country** | **Design** | **Setting, recruitment population and sample** | **Data collection** | **Analysis** | **Outcome /Findings** |
| --- | --- | --- | --- | --- | --- |
| Edin, K.E.  (2010)  Sweden | Qualitative descriptive study | Coordinators at three crisis centres at different locations recruited women who had at some stage received support at their centre. Nine women who had experienced IPV during at least one pregnancy participated. The study included a total of 14 pregnancies. | In-depth interviews performed by the first author. | Narrative analysis | Descriptions of experiences: Finding no support or sympathy from their partner during pregnancy, the women felt lonely, isolated, unhappy, sad, tired, and powerless. They described how their partners controlled and oppressed them as if they owned both them and their pregnancies. The women often felt extremely stressed and frightened during pregnancy. One described that her partner threatened her with serious consequences if she did not have an abortion. One described how her partner repeatedly told her how disgusting she was when pregnant and that she was a fat, ugly monster; he felt ashamed and did not want to be seen with her. The women seemed to successively accustom themselves to the situation and revealed their use of repressive mechanisms to shut out the pain, calm down, and trying to avoid transferring their stress to the baby in tense situations. To endure this strange day-today life, one woman said that she turned into a zombie, living in her and her baby’s own little world. The women used various strategies to enable them to endure quite conflicting feelings and, on one hand, believe that the violence was their own fault as well as, on the other hand, see it as totally wrong. |
| Engnes, K.  (2012)  Norway | Phenomenological approach | Women who had contacted the organisation Alternative to Violence (ATV) because they needed support. Total 5 women whereof 2 during pregnancy 3 after birth was recruited to the study. | In-depth interviews performed by the first author. | Analys according to Dahlberg et al. (2008) and Giorgi (2009) | The essential structure shows that IPV during pregnancy is characterized by difficult *existential choices* related to *ambivalence*. Five constituents further explain the essential structure: living in unpredictability, violence is living in the body, losing oneself, feeling lonely and being pregnant leads to change. The findings show effect of violence on women during pregnancy in depth. The body felt physically exhausted and powerless, which contributed to the women being unable to resist the violence. The violence is living in the body is also related to the fact that the foetus is living inside the woman’s body. The women were humiliated and degraded through neglect, scolding, hurtful remarks about their behaviour and appearance as well as being made a fool of in different ways. Some men checked the mobile phone to monitor who the women had been talking to. Being exposed to violence leads to experiences of being isolated and lonely. |
| Finnbogadóttir, H.  (2014b)  Sweden | A Grounded theory approach | Women recruited by two welfare officers working at shelter or through an announcement posted at two emergency wards for women. Recruitment performed between December 2011-May 2012.  Total 10 women aged  21–44 years with experience of IPV during pregnancy (survivors) who were separated from the perpetrator was recruited. The women came from different social classes and the age of the women’s youngest child ranged from 5 months to 4 years. | In-depth interviews performed by the first author. Interviews lasted between 49 minutes to 3h and 20 minutes. | Classical Grounded theory according to Glaser (2010) | Core Category: ‘Struggling to survive for the sake of the unborn baby’ The survivors deal with constant fear and violence during their pregnancy and are emotionally overloaded. They worry about whether the noise and abuse they are exposed to can affect the pregnancy and the unborn baby. The entire pregnancy revolves around not making ‘that person’ upset or mad and to survive despite the perpetrator’s impulsive anger. A deliberate choice is to stay in the relationship despite the abuse, and to avoid exposing the unborn baby to the additional stress that might be provoked by divorce proceedings, custody, and support issues, etc. Step by step the survivors adapted to the perpetrator to avoid brawls, fights, and insults because they sought to protect the unborn baby.  Categories. ‘Trapped in the situation’ demonstrates how the pregnant women feel when trapped in the relationship and cannot find their way out. ‘Exposed to mastery’ demonstrates the destructive togetherness whereby the perpetrator’s behaviour jeopardizes the safety of the woman and the  unborn child. ‘Degradation process’ demonstrates the survivor’s experience of gradual degradation because of the relationship with the perpetrator. |
| Kristinsdóttir, Á.  (2010)  Iceland (in Icelandic) | Phenomenological approach | Twelve Icelandic women,  aged 19 -72, mean age 37 ½, with a history of domestic abuse participated in the study and were interviewed once or twice, in all 15 interviews. Mean age when IPV started was 19,5 years. Average number of years in an abusive relationship 10,1 years. | In-depth interviews performed by the first author. Interviews lasted from 40 to 80 minutes. | Thematic analysis according to the Vancouver-School of Doing Phenomenology (Halldorsdottir, 2000) | The abuse started as soon as the relationship began, first emotionally then physically, sexually and even financially. All the women felt they didn’t have any respect in their homes but felt ashamed because of the abuse. When they got pregnant the situation worsened and they felt that they were stuck in the relationship. They experienced lack of consideration sexually and even violent sex. The women still have traumatic flashbacks and nightmares even many years after the abusive relationship has ended and they have suffered from anxiety and depression as well as lack of self-confidence. All their children witnessed the abuse and were seriously affected.  The study findings regarding the consequences of the IPV during pregnancy include: Constant stress, fear, fright, and anxiety; depression and great distress; increased violence during pregnancy; postpartum depression; broken self-confidence and decreased self-esteem; heavy flashbacks, difficult memories, and nightmares; loneliness and isolation; guilt and shame; severe physical symptoms and eating disorders; feeling they cannot stand by themselves; difficulty trusting others; strong feelings of rejection. |

Abbreviation list: ATV = Alternative to Violence, IPV = Intimate partner violence.
