# Supplementary material for: The Consequences of A History of Violence on Women’s Pregnancy and Childbirth in the Nordic Countries: A Scoping Review
Source: Trauma Violence Abuse. 2024 May 28;25(5):3555–70. doi: 10.1177/15248380241253044 (PMC11545221; doi:10.1177/15248380241253044)
Supplement: sj-docx-6-tva-10.1177_15248380241253044 – Supplemental material for The Consequences of A History of Violence on Women’s Pregnancy and Childbirth in the Nordic Countries: A Scoping Review [file sj-docx-6-tva-10.1177_15248380241253044.docx]

**Table S4.** List of excluded studies

|  | **Authors, year** | **DOI** | **Reason for exclusion*** |
| --- | --- | --- | --- |
| 1 | Byrskog et al. (2016) | 10.1016/j.midw.2016.05.009. | a |
| 2 | Bø Vatnar and Bjørkly (2010) | 10.1177/0886260508329129. | a |
| 3 | Ebeling et al. (2004) | 10.1111/j.07307659.2004.00285.x. | a |
| 4 | Edin and Nilsson (2013) | 10.3402/gha.v6i0.20984. | b |
| 5 | Engnes et al. (2013) | 10.1111/j.1471-6712.2012.01073.x. | a |
| 6 | Hedin and Jansson (2000) | 10.1080/j.1600-0412.2000.079008625.x. | b |
| 7 | Lepistö el al. (2017) | 10.1111/jocn.13602. | b |
| 8 | Rådestad (2004) | 10.1111/j.0730-7659.2004.00285.x. | a |
| 9 | Tomasdóttir (2016) | 10.1080/02813432.2016.1249060 | b |
| 10 | Walter et al. (2021) | 10.2196/28680. | a |

*a) not within the scope b) not a perinatal outcome
