# Supplementary material for: The Consequences of A History of Violence on Women’s Pregnancy and Childbirth in the Nordic Countries: A Scoping Review
Source: Trauma Violence Abuse. 2024 May 28;25(5):3555–70. doi: 10.1177/15248380241253044 (PMC11545221; doi:10.1177/15248380241253044)
Supplement: sj-rtf-1-tva-10.1177_15248380241253044 – Supplemental material for The Consequences of A History of Violence on Women’s Pregnancy and Childbirth in the Nordic Countries: A Scoping Review [file sj-rtf-1-tva-10.1177_15248380241253044.rtf]

		
Appendix A

 	

Search Strategy from ProQuest
2022 March 13 13:47
Medline

 	


Search StrategySearch Strategy

Set#	Searched for	Databases	Results	
S1	MESH.EXACT("Norway") OR MESH.EXACT("Greenland") OR MESH.EXACT("Finland") OR MESH.EXACT("Iceland") OR MESH.EXACT("Scandinavian and Nordic Countries") OR MESH.EXACT("Svalbard") OR MESH.EXACT("Sweden") OR MESH.EXACT("Denmark")	MEDLINE®	215351	
S2	ti,ab,if(Nordic* OR Scandinavia* OR Norway* OR norwegian* OR Svalbard* OR Denmark* OR danish OR danes OR Sweden* OR swedish OR swedes OR Finland* OR finnish OR finns OR Greenland* OR Iceland* OR "Faroe Island" OR "Faroe Islands" OR Åland OR Aland)	MEDLINE®	231841	
S3	(MESH.EXACT("Norway") OR MESH.EXACT("Greenland") OR MESH.EXACT("Finland") OR MESH.EXACT("Iceland") OR MESH.EXACT("Scandinavian and Nordic Countries") OR MESH.EXACT("Svalbard") OR MESH.EXACT("Sweden") OR MESH.EXACT("Denmark")) OR ti,ab,if(Nordic* OR Scandinavia* OR Norway* OR norwegian* OR Svalbard* OR Denmark* OR danish OR danes OR Sweden* OR swedish OR swedes OR Finland* OR finnish OR finns OR Greenland* OR Iceland* OR "Faroe Island" OR "Faroe Islands" OR Åland OR Aland)	MEDLINE®
These databases are searched for part of your query.	314086	
S4	MESH.EXACT("Pregnancy") OR MESH.EXACT("Pregnant Women") OR MESH.EXACT("Parturition") OR MESH.EXACT("Perinatal Care") OR MESH.EXACT("Postnatal Care") OR MESH.EXACT("Prenatal Care") OR MESH.EXACT("Postpartum Period") OR MESH.EXACT("Maternal-Child Health Services") OR MESH.EXACT("Maternal-Child Health Centers") OR MESH.EXACT("Maternal-Child Nursing") OR MESH.EXACT("Maternal Health") OR MESH.EXACT("Maternal Health Services") OR MESH.EXACT("Midwifery") OR MESH.EXACT("Nurse Midwives") OR MESH.EXACT("Child Health Services")	MEDLINE®	988136	
S5	ti,ab,if(pregnan* OR "expectant mother" OR "expectant mothers" OR "childbearing woman" OR "childbearing women" OR "childbearing year" OR Childbirth OR "child birth" OR "child births" OR "child's birth")	MEDLINE®	575502	
S6	ti,ab,if(antenatal* OR prenatal* OR antepartum* OR perinatal* OR postpartum* OR postnatal*)	MEDLINE®	355504	
S7	ti,ab,if(midwi* OR "Maternal child" OR "Maternal health" OR "Maternal healthcare" OR "Maternity care" OR "Maternity service" OR "Maternity services" OR "child healthcare" OR "child health care" OR "child health service" OR "child health services")	MEDLINE®	46502	
S8	(MESH.EXACT("Pregnancy") OR MESH.EXACT("Pregnant Women") OR MESH.EXACT("Parturition") OR MESH.EXACT("Perinatal Care") OR MESH.EXACT("Postnatal Care") OR MESH.EXACT("Prenatal Care") OR MESH.EXACT("Postpartum Period") OR MESH.EXACT("Maternal-Child Health Services") OR MESH.EXACT("Maternal-Child Health Centers") OR MESH.EXACT("Maternal-Child Nursing") OR MESH.EXACT("Maternal Health") OR MESH.EXACT("Maternal Health Services") OR MESH.EXACT("Midwifery") OR MESH.EXACT("Nurse Midwives") OR MESH.EXACT("Child Health Services")) OR ti,ab,if(pregnan* OR "expectant mother" OR "expectant mothers" OR "childbearing woman" OR "childbearing women" OR "childbearing year" OR Childbirth OR "child birth" OR "child births" OR "child's birth") OR ti,ab,if(antenatal* OR prenatal* OR antepartum* OR perinatal* OR postpartum* OR postnatal*) OR ti,ab,if(midwi* OR "Maternal child" OR "Maternal health" OR "Maternal healthcare" OR "Maternity care" OR "Maternity service" OR "Maternity services" OR "child healthcare" OR "child health care" OR "child health service" OR "child health services")	MEDLINE®
These databases are searched for part of your query.	1259757	
S9	MESH.EXACT("Intimate Partner Violence") OR MESH.EXACT("Spouse Abuse") OR MESH.EXACT("Domestic Violence") OR MESH.EXACT("Gender-Based Violence") OR MESH.EXACT("Violence") OR MESH.EXACT("Exposure to Violence") OR MESH.EXACT("Rape") OR MESH.EXACT("Stalking") OR MESH.EXACT("Physical Abuse") OR MESH.EXACT("Battered Women") OR MESH.EXACT("Sex Offenses") OR MESH.EXACT("Social Isolation") OR MESH.EXACT("Emotional Abuse") OR MESH.EXACT("Coercion")	MEDLINE®	82996	
S10	ti,ab,if((partner* OR spous* OR husband* OR wife OR wives OR marital OR marriage* OR married OR domestic* OR family OR women* OR woman* OR pregnan* OR "expectant mother*") NEAR/3 (abuse* OR abusive* OR violen* OR victim* OR battere* OR battering OR beat* OR threat*))	MEDLINE®	35481	
S11	ti,ab,if((Psychological OR Emotional OR Physical OR Verbal OR Sexual) NEAR/1 (abuse* OR violen* OR aggress*))	MEDLINE®	32870	
S12	ti,ab,if(IPV OR "Gender-based violen*" OR "interpersonal violen*" OR "survivor* of violen*" OR "Social isolation" OR stalker OR stalking OR "controlling behavior" OR "controlling behaviors" OR "controlling behaviour" OR "controlling behaviours" OR Rape OR "exposure to violen*" OR "Sex offense" OR "Sex offenses" OR Coercion OR coercive OR "intimate terrorism")	MEDLINE®	38558	
S13	(MESH.EXACT("Intimate Partner Violence") OR MESH.EXACT("Spouse Abuse") OR MESH.EXACT("Domestic Violence") OR MESH.EXACT("Gender-Based Violence") OR MESH.EXACT("Violence") OR MESH.EXACT("Exposure to Violence") OR MESH.EXACT("Rape") OR MESH.EXACT("Stalking") OR MESH.EXACT("Physical Abuse") OR MESH.EXACT("Battered Women") OR MESH.EXACT("Sex Offenses") OR MESH.EXACT("Social Isolation") OR MESH.EXACT("Emotional Abuse") OR MESH.EXACT("Coercion")) OR ti,ab,if((partner* OR spous* OR husband* OR wife OR wives OR marital OR marriage* OR married OR domestic* OR family OR women* OR woman* OR pregnan* OR "expectant mother*") NEAR/3 (abuse* OR abusive* OR violen* OR victim* OR battere* OR battering OR beat* OR threat*)) OR ti,ab,if((Psychological OR Emotional OR Physical OR Verbal OR Sexual) NEAR/1 (abuse* OR violen* OR aggress*)) OR ti,ab,if(IPV OR "Gender-based violen*" OR "interpersonal violen*" OR "survivor* of violen*" OR "Social isolation" OR stalker OR stalking OR "controlling behavior" OR "controlling behaviors" OR "controlling behaviour" OR "controlling behaviours" OR Rape OR "exposure to violen*" OR "Sex offense" OR "Sex offenses" OR Coercion OR coercive OR "intimate terrorism")	MEDLINE®
These databases are searched for part of your query.	135927	
S14	((MESH.EXACT("Norway") OR MESH.EXACT("Greenland") OR MESH.EXACT("Finland") OR MESH.EXACT("Iceland") OR MESH.EXACT("Scandinavian and Nordic Countries") OR MESH.EXACT("Svalbard") OR MESH.EXACT("Sweden") OR MESH.EXACT("Denmark")) OR ti,ab,if(Nordic* OR Scandinavia* OR Norway* OR norwegian* OR Svalbard* OR Denmark* OR danish OR danes OR Sweden* OR swedish OR swedes OR Finland* OR finnish OR finns OR Greenland* OR Iceland* OR "Faroe Island" OR "Faroe Islands" OR Åland OR Aland)) AND ((MESH.EXACT("Pregnancy") OR MESH.EXACT("Pregnant Women") OR MESH.EXACT("Parturition") OR MESH.EXACT("Perinatal Care") OR MESH.EXACT("Postnatal Care") OR MESH.EXACT("Prenatal Care") OR MESH.EXACT("Postpartum Period") OR MESH.EXACT("Maternal-Child Health Services") OR MESH.EXACT("Maternal-Child Health Centers") OR MESH.EXACT("Maternal-Child Nursing") OR MESH.EXACT("Maternal Health") OR MESH.EXACT("Maternal Health Services") OR MESH.EXACT("Midwifery") OR MESH.EXACT("Nurse Midwives") OR MESH.EXACT("Child Health Services")) OR ti,ab,if(pregnan* OR "expectant mother" OR "expectant mothers" OR "childbearing woman" OR "childbearing women" OR "childbearing year" OR Childbirth OR "child birth" OR "child births" OR "child's birth") OR ti,ab,if(antenatal* OR prenatal* OR antepartum* OR perinatal* OR postpartum* OR postnatal*) OR ti,ab,if(midwi* OR "Maternal child" OR "Maternal health" OR "Maternal healthcare" OR "Maternity care" OR "Maternity service" OR "Maternity services" OR "child healthcare" OR "child health care" OR "child health service" OR "child health services")) AND ((MESH.EXACT("Intimate Partner Violence") OR MESH.EXACT("Spouse Abuse") OR MESH.EXACT("Domestic Violence") OR MESH.EXACT("Gender-Based Violence") OR MESH.EXACT("Violence") OR MESH.EXACT("Exposure to Violence") OR MESH.EXACT("Rape") OR MESH.EXACT("Stalking") OR MESH.EXACT("Physical Abuse") OR MESH.EXACT("Battered Women") OR MESH.EXACT("Sex Offenses") OR MESH.EXACT("Social Isolation") OR MESH.EXACT("Emotional Abuse") OR MESH.EXACT("Coercion")) OR ti,ab,if((partner* OR spous* OR husband* OR wife OR wives OR marital OR marriage* OR married OR domestic* OR family OR women* OR woman* OR pregnan* OR "expectant mother*") NEAR/3 (abuse* OR abusive* OR violen* OR victim* OR battere* OR battering OR beat* OR threat*)) OR ti,ab,if((Psychological OR Emotional OR Physical OR Verbal OR Sexual) NEAR/1 (abuse* OR violen* OR aggress*)) OR ti,ab,if(IPV OR "Gender-based violen*" OR "interpersonal violen*" OR "survivor* of violen*" OR "Social isolation" OR stalker OR stalking OR "controlling behavior" OR "controlling behaviors" OR "controlling behaviour" OR "controlling behaviours" OR Rape OR "exposure to violen*" OR "Sex offense" OR "Sex offenses" OR Coercion OR coercive OR "intimate terrorism"))	MEDLINE®
These databases are searched for part of your query.	270	


 	
Database copyright Ó 2022 ProQuest LLC. All rights reserved.
Terms and Conditions    Contact ProQuest 
